# Artificial intelligence and machine learning in sports medicine: mapping clinical tasks and assessing clinical maturity - a scoping review

**DOI:** 10.1186/s12911-026-03615-w

**Published:** 2026-06-12

**Authors:** Jakob Lindskog, Kristian Heder Ternell, Yinan Yu, Ida Lindman, Kristian Samuelsson, Eric Hamrin Senorski

**Affiliations:** 1 Unit of Physiotherapy, Department of Health and Rehabilitation, Institute of Neuroscience and Physiology, Sahlgrenska Academy, University of Gothenburg, Box 455, Gothenburg, SE-405 30 Sweden; 2 Sahlgrenska Sports Medicine Center, Sahlgrenska Academy, Gothenburg, Sweden; 3 Department of Orthopaedics, Institute of Clinical Sciences, The Sahlgrenska Academy, University of Gothenburg, Gothenburg, Sweden; 4 Department of Computer Science and Engineering, Chalmers University of Technology and University of Gothenburg, Gothenburg, Sweden; 5 General Practice / Family Medicine, School of Public Health and Community Medicine, Institute of Medicine, Sahlgrenska Academy, University of Gothenburg, Gothenburg, Sweden; 6 Development & Innovation, Primary Health Care, Region Västra Götaland, Gothenburg, Sweden; 7 Department of Orthopaedics, Sahlgrenska University Hospital, Mölndal, Sweden

## Abstract

**Background:**

Artificial intelligence (AI) and machine learning (ML) are rapidly transforming the medical field. The aim of this review was to outline the current scientific state of AI and ML application in sports medicine, evaluate the level of clinical validation and readiness for implementation, and identify key priorities to guide future advancements and implementation into injury risk assessment, diagnosis, rehabilitation and clinical decision-making in sport medicine.

**Methods:**

A scoping review was conducted with a literature search performed on February 5, 2026, using the MEDLINE, EMBASE and Web of Science databases which targeted AI or ML application on individuals within a sports medicine context.

**Results:**

Of 8,677 studies, 97 studies were included. Most research covered orthopaedics (70.1%) and neurology (18.6%), where AI was applied for injury prediction, diagnostic image analysis, and recovery estimation. Predictive and estimation models were the dominant application (57.7%). Reported discriminative performance was frequently high. However, the majority of studies relied on retrospective datasets and internal validation. Calibration reporting was uncommon, and prospective workflow integration was rare, with a single study attempting an interventional prevention strategy. Substantial heterogeneity in modelling approaches, data inputs, and outcomes definitions was observed.

**Conclusion:**

Although AI and ML applications in sports medicine frequently demonstrate strong within-sample performance, most remain in early-stage development. Currently, these tools should be viewed as supportive adjuncts rather than autonomous decision-making systems.

**Key Terms:**

AI, Predictive modeling, Diagnostic imaging, Rehabilitation, Deep learning, Return to sport

**Supplementary Information:**

The online version contains supplementary material available at 10.1186/s12911-026-03615-w.

## Introduction

Artificial intelligence (AI) and machine learning (ML) are transforming healthcare by enabling machines to effectively analyze data, recognize patterns, and aid decision-making [1]. There is research that suggests that AI and ML might outperform humans within specific healthcare domains [2–4]. For example, ML models have, based on radiographic analysis, identified patients at risk for poor outcomes after unicompartmental knee replacement with greater accuracy than surgeons [2]. Similarly, chatbots powered by large language models (LLMs) have in one study surpassed physicians in diagnostic accuracy [3]. Furthermore, AI has reduced the miss rate for colorectal neoplasia detection by half [4]. These examples highlight the potential and increasing role of AI and ML in improving healthcare outcomes.

Within sports medicine, AI and ML applications have attracted considerable interest. For example, Hu et al. [5] demonstrated that the use of a convolutional neural network (CNN) could detect anterior cruciate ligament (ACL) injury through magnetic resonance imaging (MRI) with an accuracy of 96.5%. Furthermore, Allen et al. [6] reported that a decision tree model could discriminate between early, typical and delayed recovery after sports-related concussion (SRC). Moreover, an extreme gradient boosting (XGBoost) model was used to predict level of match participation in football athletes after Achilles tendon rupture [7]. In addition, a recent scoping review found that ML models have been widely applied to support ACL injury prediction, rehabilitation monitoring and return to sport (RTS) decision-making [8]. These studies showcase the potential of AI and ML in sports medicine. However, the literature remains fragmented, with limited understanding of how applications vary across fields and whether current work has progressed beyond retrospective analyses toward prospective or interventional use. Furthermore, it remains unclear whether existing AI and ML applications have reached sufficient developmental and clinical maturity to inform decision-making in sports medicine.

The aim of this review was to outline the current scientific state of AI and ML application in sports medicine, evaluate the level of clinical validation and readiness for implementation, and identify key priorities to guide future advancements and implementation into injury risk assessment, diagnosis, rehabilitation and clinical decision-making in sport medicine.

## Method

### Protocol and registration

As this was conducted as a scoping review with the purpose to map a rapidly growing field a formal review protocol was not considered needed.

### Eligibility criteria

To be included in this review, papers needed to be written in English, published year 2000 or later, and were required to report on the use of AI or ML within the context of sports medicine. Peer-reviewed papers on all levels of evidence in accordance with the Oxford classification were included. All empirical study designs, including quantitative, qualitative, mixed-methods, case studies, and pilot studies, were considered with no restrictions with regards to cohort size or patient characteristics. Reviews, conference abstracts, commentaries (editorials, opinion pieces), system proposals (frameworks, protocols, datasets), articles without full-text availability, and pre-prints were excluded. System proposals were excluded, as the scope of this review was limited to studies that investigated the application of AI/ML within sports medicine.

### Information sources

This study was conducted and presented in accordance with the Preferred Reporting Items for Systematic Reviews and Meta-Analyses extension for Scoping Reviews (PRISMA-ScR) checklist [9]. A scoping review approach was performed due to the heterogeneity of AI methods, outcomes, and study designs, which made a quantitative synthesis and detailed risk of bias assessment less feasible. A literature search was planned in collaboration with and was executed out on 2026–02-05 by medical university librarians, with expertise in electronic database searching. The search strategies were peer reviewed by another senior medical university librarian prior to execution in accordance with the PRESS Checklist [10]. No additional manual searching of references list or grey literature was conducted after the primary database search. The literature search included the MEDLINE, EMBASE, and Web of Science databases, to provide comprehensive coverage of sports medicine research relevant to the clinical scope of this study.

### Search

The initial search strategy was developed to identify studies which involved the use of AI or ML technologies in the context of sports medicine. The search targeted two core elements. First, the population: Individuals within a sports medicine context (athletes, physically active individuals or patients with or at risk of sports related injury). Second, the concept: Utilization of AI- or ML-based technologies, tools, or platforms intended to support the prevention, diagnosis, rehabilitation, or RTS decision-making within sports medicine. The broad search strategy was adopted to comprehensively map the diversity of AI and ML applications in sports medicine. The complete search strategy can be found in the Supplementary information.

### Selection of sources of evidence

The screening process was performed with the Rayyan reference management platform (rayyan.ai) [11]. Screening of studies was performed by two independent reviewers (JL and KHT) starting with title and abstract screening followed by full-text evaluation. At all stages, any differences were resolved by mutual agreement. For title and abstract screening, agreement between the authors was assessed with Cohen’s Kappa coefficient. Inter-rater reliability for full-text screening was not formally quantified; disagreements were resolved through discussion and consensus between reviewers.

### Data charting process

Draft data charting tables were developed in Microsoft Excel (Version 16, Microsoft Corporation, Remond, WA, USA) to record extracted data from the included articles, guided by the scoping review research statement. The first two authors (JL and KHT) performed the data charting process.

### Data items

Study characteristics extracted included: bibliographic details (author(s), year of publication, and scientific journal), study characteristics (medical field, and country of origin), field of population (sport, sample size, age, study participant sex), AI-/ML application, data sources and input variables, output variables (outcomes), AI-/ML model(s) used, and key findings. Medical field classification was based on the primary clinical focus of each study. Studies were categorized as orthopaedics when the AI or ML application addressed musculoskeletal pathology, surgical outcomes, or rehabilitation related to orthopaedic conditions.

The AI applications were categorized into four mutually exclusive application groups based on the primary aim of the AI component for each study. This categorization was not based on a single established or universally accepted classification framework; however, was pragmatically developed for the purpose of synthesizing the heterogeneous literature. Categories were defined a priori as follows: 1) predictive and estimation models, in which algorithms were primarily used to predict outcomes, risks, or clinical parameters; 2) classification and pattern recognition models, which focused on classification, clustering, feature extraction, or anomaly detection; 3) diagnostic and detection models, which aimed to identify diseases, conditions, or abnormalities; and 4) reasoning and summarization assistance models, where LLMs were central. When a study could plausibly fit more than one category, it was assigned to the group that best reflected the dominant role of the AI-/ML component.

### Critical appraisal of individual sources of evidence

Due to the exploratory and descriptive nature of scoping reviews, and the heterogeneity of included studies, risk of bias and study quality assessments were not feasible and did not influence scoping review outcomes.

### Synthesis of results

The results from the included studies were synthesized descriptively with a narrative approach, supported by tables and figures where appropriate [9]. Data charted from each study was grouped and summarized in accordance with key themes. These included: medical fields studied, AI-/ML applications, years and countries of publication, study populations, AI-/ML models used in general, with more detailed subgroup analyses performed for orthopaedics and neurology due to the higher number of included studies in these fields, and lastly, the overall model development stage.

No meta-analysis or quantitative pooling was conducted due to the heterogeneity of study designs, AI-/ML approaches, and reported outcomes. Instead, findings were mapped to highlight the breadth of research activity, common areas of application, and gaps in the literature.

## Results

**Fig. 1:**
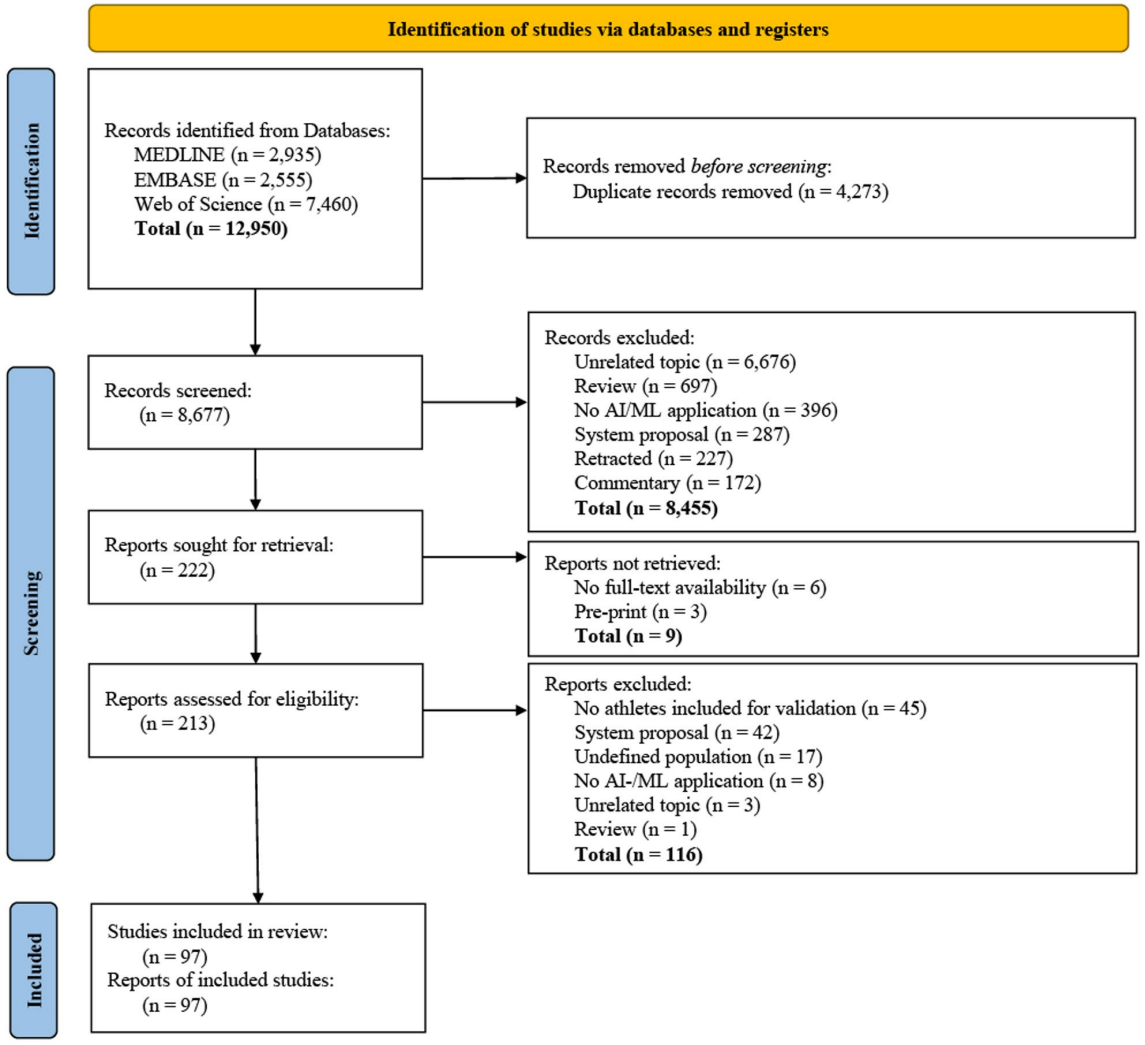
PRISMA flowchart for the inclusion process. AI = Artificial intelligence, ML = Machine learning, n = number

In total, 8,677 records were identified, of which 97 were included (Fig. 1). For title and abstract screening, the Cohen’s Kappa coefficient was calculated to 0.877, which suggests near perfect agreement. Of the included studies, the medical fields represented were as follows: orthopaedics (*n* = 68, 70.1%), neurology (*n* = 18, 18.6%), radiology (*n* = 3, 3.1%), cardiology and cardiopulmonary (*n* = 2, 2.1%), nephrology (*n* = 1, 1.0%), odontology (*n* = 1, 1.0%), endocrinology (*n* = 1, 1.0%) and various (self-reported participation-restricting injuries [non-diagnosis-specific]; sports rehabilitation and digital health) (*n* = 3, 3.1%) (Table 1). Within orthopaedics, AI was primarily applied for injury prediction, outcome estimation, and rehabilitation monitoring, particularly concerning lower-extremity and ACL-related injuries. In neurology, models predominantly focused on SRC management, including classification of SRC severity and prediction of recovery duration. Radiology studies used AI for automated image optimization, detection of ligament injuries and bone marrow lesions, while the remaining medical fields involved isolated applications in injury-risk estimation (cardiopulmonary and cardiology), acute physiological responses (nephrology), dental injury prediction (odontology), and low bone mineral density (endocrinology).

**Table 1:** Overview of included studies

Author	Year	Journal	Country	Medical field	Field of population	Sample size, n	Age, mean years or range	Sex, males %	AI application	AI outcome	Model(s)	Key findings	Data used
Abasi et al.[12]	2025	BioData Minin	Iran	Cardiopulmonary	Elite football	256	NR	NR	Predictive & Estimation models	Prediction of reinjury risk	SVM, CatBoost, RF, XGBoost	CatBoost; Acc: 0.9138, F1: 0.9148; SVM: AUC: 0.9725	Cardiopulmonary data
Allen et al.[6]	2023	Journal of Neurosurgery: Pediatrics.	USA	Neurology	Pediatric athletes	493	15.7	68	Predictive & Estimation models	Prediction of early (≤14), typical (15–27), delayed (≥28) recovery time (days) from SRC	DT	AUC: 0.80, Youden: 0.44. Sen: >0.90 (Classified early recovery)	Demographics, post-SRC symptom scales, time-to-clinic presentation, concussion history, presence of defined symptom clusters
Aoyag et al.[13]	2021	Spine	Japan	Orthopaedics	Junior high-school athletes	223	13.5	72	Diagnostic & Detection models	Distinguish lumbar spondylolysis from non-specific low back pain	CART	Sen: 0.64, Spec: 0.92, AUC: 0.79	Demographics, school grades, symptom onset time, history of lower-back pain, pre-existing conditions and anthropometry
Ayala et al.[14]	2019	International Journal of Sports Medicine	Spain	Orthopaedics	Professional football	96	NR	100	Predictive & Estimation models	Prediction of risk factors of hamstring injury	DT	AUC: 0.837, Sen: 0.778, Spec: 0.838	Preseason: psychological, neuromuscular, and demographical data
Bazarian et al.[15]	2021	JAMA Network Open	USA	Neurology	Athletes	580	19.5	54	Classification & Pattern recognition	Classification of SRC based on EEG	Genetic algorithm	Sen: 0.860, Spec 0.708, NPV: 0.901, PPV: 0.620, AUC: 0.89	EEG, cognitive tests, symptom inventories
Bergeron et al.[16]	2019	Medicine & Science in Sports & Exercise	USA	Neurology	High-school football	2004	NR	NR	Predictive & Estimation models	Estimation of symptom resolvment after SRC	NB, SVM, 5-nearest neighbours, DT, RF, MLP, radial basis function network	NB and RF with 100 or 500 trees: AUC: 0.656–0.742	Symptom and recovery data
Briand et al.[17]	2022	Frontiers in Sports & Active Living	Canada	Various (orthopaedics and neurology)	Short-track speed skaters	11	21	0	Predictive & Estimation models	Prediction of injury	RF	Sen: 0.5, Spec 0.7	Longitudinal: training load, physiological, neuromuscular, psychological well-being, heart rate variability and injury history data
Calderon-Diaz et al.[18]	2023	Sensors	Chile	Orthopaedics	Professional football	110	NR	100	Predictive & Estimation models	Prediction of muscle injury	DT, discriminant methods, NB, SVM, KNN, NN, XGBoost	XGBoost: Prec: 78%.	Biomechanical and muscle performance data
Cao et al.[19]	2008	IEEE Transactions on Neural Systems & Rehabilitation Engineering	USA	Neurology	Rugby and American football	61	20	44	Classification & Pattern recognition	Classification of residual functional deficit	SVM	Acc: 77.1%, Sen: 80.0%, Spec: 75.0%	EEG
Castellanos et al.[20]	2021	Sports Medicine	USA	Neurology	US Military cadettes	15682	19	65	Predictive & Estimation models	Prediction of SRC risk	SVM	AUC: 0.73	Baseline demographic, clinical, cognitive and behavioral data
Chen et al.[21]	2022	Computational & Mathematical Methods in Medicine	China	Orthopaedics	Basketball	935	20	76	Classification & Pattern recognition	Classification of thoracolumbar vertebral fractures (ABC)	Deeplearning: Faster RCNN	Acc: 86.4%, Cohen’s kappa: 0.850	CT images
Chu et al.[22]	2022	Annals of Physical & Rehabilitation Medicine	USA	Neurology	Youth athletes	655	13.7 (male), 14.0 (female)	55	Predictive & Estimation models	Prediction of SRC recovery	CatBoost, DT, elastic net, RF, XGBoost, TabNet	CatBoost: AUC: 0.8 (males), and 0.78 (females)	Preinjury risk factors, injury severity measures, post-SRC functional and symptom data
Dandrieux et al.[23]	2025	BMJ Open Sport & Exercise Medicine	France	Various	Track and field	112	34	62	Predictive & Estimation models	Investigate association between injury risk estimation and injury burden	Negative binomial regression	AUC: 0.63	Longitudinal: training activity, psychological state, sleep quality, and self-reported injury status
De la Fuente et al.[24]	2023	Science & Medicine in Football	Chile	Orthopaedics	Football	21	22.5	0	Classification & Pattern recognition	Clustering to determine risk profiles based on biomechanical properties	umap	3 clusters of biomechanical properties	Biomechanical data
de Leeuw et al.[25]	2022	European Journal of Sport Science	Netherlands	Orthopaedics	Volleyball	10	27	100	Predictive & Estimation models	Prediction of overuse injuries	Subgroup discovery	Jump load was an important predictor for 70% of players	Longitudinal: training load, subjective wellness reports and overuse symptom questionnaires
DiCesare et al.[26]	2020	Annals of Biomedical Engineering	USA	Neurology	Football	20	16	0	Classification & Pattern recognition	Classification of sub-SRC impact exposure	XGBoost	Acc: 83.5%	Wearable sensor data, video-verified head impact recordings and MRIs
Diniz et al.[7]	2022	Knee Surgery, Sports Traumatology, Arthroscopy	Various	Orthopaedics	Football	209	28.3	100	Predictive & Estimation models	Prediction of level of match participation	Clustering, XGBoost	XGBoost: AUC: 0.81	Match participation and performance data
Diniz et al.[27]	2024	Knee Surgery, Sports Traumatology, Arthroscopy	Various	Orthopaedics	Football	236	26.6	100	Predictive & Estimation models	Cross-validation of identified ACL injury mentions	OpenAI’s GPT-4o mini	Sen: 88.4%, Spec: 99.3%	Publicly available textual and database data
Elkin et al.[28]	2018	Applied Clinical Informatics	USA	Orthopaedics	Mixed	469	44	50	Diagnostic & Detection models	Diagnosis of knee injury	Bayesian and heuristic model	Specificity-based Bayesian model significantly outperformed heuristic model	Patient-reported questionnaire
Eskofier et al.[29]	2012	Computer Methods in Biomechanics & Biomedical Engineering	Canada	Orthopaedics	Runners	80	41.1 (male), 36.0 (female)	50	Classification & Pattern recognition	Classification of participants with or without patellofemoral pain syndrome	AdaBoost	Acc: 100%.	Biomechanical data
Evans et al.[30]	2024	PLoS ONE	UK	Orthopaedics	Rugby	36	20.7	100	Classification & Pattern recognition	Classification of non-contact lower limb injuries risk factors	Bayesian pattern recognition and assessed by means of: NB, J48 DT, SVM, KNN	AUC 0.76 (severe non-contact lower limb), 0.70 (non-contact lower limb), and 0.71 (non-contact ankle)	Longitudinal: training load, performance test results, musculoskeletal screening metrics and injury history
Farhadian et al.[31]	2020	BMC Sports Science, Medicine and Rehabilitation	Iran	Odontology	Pediatric athletes	356	11.3 (injured), 10.6 (uninjured)	54	Predictive & Estimation models	Prediction of dental injury	RF	Acc: 89.3%	Demographic and behavioral data
Ferris et al.[32]	2021	American Journal of Sports Medicine	USA	Neurology	Collegiate athletes	388	19.9	63	Diagnostic & Detection models	Diagnosis of SRC	AdaBoost	Increased diagnostic accuracy by 4.4% to AUC: 0.848, and increased Sen by 9%	Multimodal concussion assessment data
Freitas et al.[33]	2025	PLoS ONE	Portugal	Orthopaedics	Professional football	34	26.3	100	Predictive & Estimation models	Prediction of non-contact injuries in footballers	SVM, Feedforward NN, AdaBoost	SVM: Acc: 74%, Sen: 71%, Spec: 74%	Wearable GPS data
Garcia et al.[34]	2019	Journal of Neurotrauma	USA	Neurology	Athletes and military	24561	19.3	58	Predictive & Estimation models	Prediction of SRC levels	CART	Sen: 91.07% to 97.40%	Concussion assessment and demographic data
Gaudet et al.[35]	2019	Journal of Science & Medicine in Sport	Canada	Orthopaedics	Swimmers and handball	34	21.7	0	Classification & Pattern recognition	Clustering to determine shoulder injury based on subjective outcomes	K-mean clustering	Sen: 86%, Spec: 100%, diagnostic OR: 229.67 (KJOC). Sen: 86%, Spec: 37%, diagnostic OR: 3.53 (CKQUEST)	Functional performance and self-reported clinical assessment data
Giorgino et al.[36]	2024	Diagnostics	Italy	Orthopaedics	NA	NA	NA	NA	Reasoning & summarization assistance	Use of an LLM for patient education	Google Bard & ChatGPT-3.5	Both models show good promise in patient education	Text-based conversation responses
Girard et al.[37]	2025	Knee Surgery, Sports Traumatology, Arthroscopy	Canada	Orthopaedics	Adolescents with and without ACL injury	134	15.3 (ACL injured), 13.8 (controls)	30 (ACL injured) 44 (controls)	Classification & Pattern recognition	Classification of ACL injury status	DT	Entire group: Acc 0.675, Sen 0.70, Spec 0.65, F1 0.684; Females: Acc 0.769; Males: Acc 0.533	Biomechanical data
Goggins et al.[38]	2022	International Journal of Sports Medicine	UK	Orthopaedics	Elite pathway cricket	17	18.2	0	Predictive & Estimation models	Prediction of injury	DT, RF	DT: AUC: 0.66. RF: AUC: 0.72	Longitudinal: training load and performance monitoring data
Gultekin et al.[39]	2025	Knee Surgery, Sports Traumatology, Arthroscopy	Turkey	Orthopaedics	NA	NA	NA	NA	Reasoning & Summarization models	Evaluation of LLM-generated ACL surgery patient education responses	ChatGPT-4o, DeepSeek R1	Both high accuracy (3.9/4) and consistency (4/4); ChatGPT more comprehensive (4.0 vs 3.2, *p* < 0.001); DeepSeek clearer (3.9 vs 3.0, *p* < 0.001) and more readable (FKGL 8.9 vs 14.2; FRES 61.3 vs 32.7)	Text-based conversation responses
Guo et al.[40]	2025	PeerJ	China	Orthopaedics	Collegiate basketball	104	20.4	100	Predictive & Estimation models	Prediction of ACL injury incidence	RF, SVM, XGBoost, LR	RF: AUC 0.80; Accuracy 0.962; XGBoost AUC 0.79; Logistic regression AUC 0.76; SVM AUC 0.66	Demographic, injury history, biomechanical and EMG data
Hecksteden et al.[41]	2023	Science and Medicine in Football	Germany	Orthopaedics	Professional football	88	24.6	100	Predictive & Estimation models	Forecasting non-contact time-loss injuries	GBoost, LR	GBoost: CV AUC 0.61; Test AUC 0.62; without screening data AUC 0.56; without upsampling AUC 0.48	Physical performance, clinical, injury history and daily training, recovery and exposure data
Henriquez et al.[42]	2020	Frontiers in Sports & Active Living	USA	Orthopaedics	Student athletes	122	19.6	59	Predictive & Estimation models	Prediction of musculoskeletal injury	RF	Acc: 79%	Biomechanical, physical performance, demographic and injury history data
Hopkingson et al.[43]	2022	European Journal of Sport Science	Various European	Orthopaedics	Rugby	246	NR	NR	Classification & Pattern recognition	Classification of injurious or no-injurious tackles	RF	Acc: 0.919, Sen: 0.995, Spec: 0.525	Video-derived tackle characteristics
Hsu et al.[44]	2022	Journal of Human Kinetics	Various	Nephrology	Ultramarathon runners	22	44	95	Predictive & Estimation models	Prediction of acute kidney injury	SVM	Sen: 90%, Spec 100%	Baseline psychological, biochemical, and body composition data
Hu et al.[5]	2025	Scientific Reports	Croatia	Radiology	Mixed	3064	NR	NR	Diagnostic & Detection models	Detection of ACL injury	CNN + modified political optimizer	Acc: 96.496%, Sen: 99.767%, Spec: 98.557%	MRI
Huang et al.[45]	2022	Frontiers in Physiology	China	Orthopaedics	Youth basketball	16	16.6	0	Predictive & Estimation models	Prediction of lower extremity non-contact injury	Fusion model, XGBoost, RF	Fusion model: Prec: 0.9932, recall: 0.9976, F2: 0.9967 (non-injured). Prec: 0.9317, recall: 0.9167, F2: 0.9171 (minimal LE NC). Prec: 0.9000, recall: 0.9000, F2: 0.9000 (mild LE NC)	Longitudinal: training load, perceived well-being, psychological responses, physical performance metrics, and injury history
Huang et al.[46]	2023	Frontiers in Physiology	China	Orthopaedics	Youth basketball	17	15	0	Predictive & Estimation models	Prediction of lower limb non-contact injury	Cost-sensitive NN	AUC: 0.8590, Prec: 0.6360, recall: 0.8700, F2: 0.7980, Brier: 0.1020	Physical fitness, physiological data: performance metrics, biochemical markers, physiological responses, and perceived exertion
Hwang et al.[47]	2025	Orthopaedic Journal of Sports Medicine	South Korea	Orthopaedics	Athletes	113	27	67	Predictive & Estimation models	Prediction of subjective function, symptoms, and psychological readiness	GBoost, SVM, LR, DT, RF	GBoost: AUC: 0.844, F1: 0.889 (Successful recovery of PASS, IKCD). RF: AUC: 0.835, F1:0.732 (PASS ACL-RSI)	Isokinetic muscle strength and y-balance test results and patient reported outcomes
Hwang et al.[48]	2024	Digital Health	South Korea	Orthopaedics	Athletes	102	30	74	Predictive & Estimation models	Prediction of return to sport after ACL reconstruction	RF, GBoost	RF: AUC: 0.952 (single leg hop), and 0.949 (Tegner activity scale). GBoost: AUC: 0.868 (single leg vertical hop)	Physical performance data: balance, and isokinetic muscle strength
Jacob et al.[49]	2022	Scientific Reports	Iceland	Neurology	Elite athletes	54	38.4	0	Classification & Pattern recognition	Classification of SRC	RF, GBoost, AdaBoost, SVM, MLP	SVM: Acc: 95.5%	EEG, EMG; heart rate, and center of pressure and concussion assessment scale (SCAT5)
Jauhiainen et al.[50]	2022	American Journal of Sports Medicine	Various	Orthopaedics	Elite football and handball	791	21	0	Predictive & Estimation models	Prediction of ACL injury	SVM linear and with imbalance handling, RF, L2-regularized LR	Linear SVM: AUC: 0.63	Preseason biomechanical and physical performance data
Jauhiainen et al.[51]	2020	International Journal of Sports Medicine	Finland	Orthopaedics	Youth basketball and floorball	314	16.0 (male), 15.4 (female)	45	Predictive & Estimation models	Prediction of injury risk	RF	AUC: 0.63	Baseline biomechanical and physical performance data and anthropometrics
Jia et al.[52]	2022	Computational Intelligence & Neuroscience	China	Orthopaedics	Gymnasts	126	15.3	0	Classification & Pattern recognition	Identification of injury through images	Fuzzy pattern recognition. NN.	Identified injury situation through images	Image data and biomechanical force analysis
Karbalaie et al.[53]	2026	Journal of Sports Sciences	Sweden	Orthopaedics	Mixed: Patients with ACL-R	107	25.2 (ACLR), 22.4 (controls)	36	Classification & Pattern recognition	Classification of high versus low fear of re-injury	CNN, LR	CNN: Acc 75.6%, F1 0.6, MCC 0.52; 8.6% higher Acc compared to LR	Biomechanical data
Kolodziej et al.[54]	2023	Scandinavian Journal of Medicine & Science in Sports	Germany	Orthopaedics	Youth elite football	56	17.2	100	Predictive & Estimation models	Prediction of lower extremity injury risk	LASSO. Leave-One-Out	LASSO: AUC: 0.63, Sen: 35%, Spec: 79%	Biomechanical, neuromuscular and postural control data
Kunze et al.[55]	2021	Journal of Bone & Joint Surgery	USA	Orthopaedics	Athletes	1118	30	32	Predictive & Estimation models	Prediction of functional Improvement	ENPLR, stochastic GBoost, RF, AdaBoost, NN, SVM	ENPLR: AUC: 0.77, intercept: 0.7, slope: 1.22, Brier: 0.14	Clinical, demographic and radiographic registry data
Kunze et al.[56]	2021	Orthopaedic Journal of Sports Medicine	USA	Orthopaedics	Mixed: Patients with ACL-R	442	29	52	Predictive & Estimation models	Prediction of clinically meaningful improvement after ACL reconstruction	Stochastic GBoost, RF, NN, SVM, AdaBoost, ENPLR	ENPLR: AUC: 0.82, intercept: 0.10, slope: 1.15, Brier: 0.068	Clinical and surgical registry data
Lipps Lene et al.[57]	2024	Journal of Experimental Orthopaedics	France	Orthopaedics	Athletes	96	21.9 (male), 21.1 (female)	64	Diagnostic & Detection models	Identification of participants with earlier knee injury	DT, MLP, XGBoost	DT and MPL: AUC: 0.94, Acc: 0.95, Prec: 1.0, Recall: 0.88, F1: 0.93	Biomechanical and psychological data
López-Valenciano et al.[58]	2018	Medicine & Science in Sports & Exercise	Spain	Orthopaedics	Professional football and handball	132	NR	100	Predictive & Estimation models	Prediction of muscle injury	C4.5 DT, SimpleCart, ADTree, RandomTree	ADTree: AUC: 0.747, Sen: 65.9%, Spec: 79.1%	Preseason demographic, psychological and neuromuscular data
Lövdal et al.[59]	2021	International Journal of Sports Physiology & Performance	Netherlands	Orthopaedics	High-level middle- and long-distance runners	74	NR	64	Predictive & Estimation models	Prediction of injury	XGBoost	AUC: 0.724 (day), and 0.678 (week)	Longitudinal training load data (GPS and subjective training feedback)
Lu et al.[60]	2022	Orthopaedic Journal of Sports Medicine	USA	Orthopaedics	Elite basketball	2103	26	100	Predictive & Estimation models	Prediction of lower extremity muscle strain/injury	XGBoost, RF, NN, SVM, elastic net penalized LR, generalized LR	XGBoost AUC: 0.840	Longitudinal player performance and historical injury data
Martínez-Gramage et al.[61]	2020	Sensors	Spain	Orthopaedics	Triathletes	19	14.6	53	Predictive & Estimation models	Prediction of running injury	RF	AUC: 0.8, Sen: 0.6, Spec: 0.8, NPV 0.7, Matthews correlation coefficient 0.4	Biomechanical, neuromuscular, and injury incidence data
Maxin et al.[62]	2024	Diagnostics	USA	Neurology	Collegiate football	93	20	100	Diagnostics & Detection models	Diagnosis of acute SRC	RF, KNN, SVM, LR (SMOTE)	Post-SMOTE RF: Acc 91%, Sen 98%, Spec 86%, AUC 0.91, F1 0.92	Smartphone-based quantitative pupillometry
McBee et al.[63]	2024	JMIR medical education	USA	Various	NA	NA	NA	NA	Reasoning & summarization assistance	LLM for interdisciplinary panel discussion on sports medicine	ChatGPT-4	Reasonably pointed to various benefits such as 24/7 support, personalized advice, automated tracking, and reminders	Text-based conversation data
Murray et al.[64]	2024	Sports & Health	USA	Neurology	Student athletes	409	20	56	Classification & Pattern recognition	Classification participants with or without SRC	LR	Single-task tests were slower in patients with SRC	Biomechanical and behavioral performance data including cognitive task response rates
Nechita et al.[65]	2025	Diagnostics	Romania	Cardiology	Youth athletes	312	7 to 17	NR	Diagnostic & Detection models	Detection of cardiovascular injury risk	RF, CNN	RF: Acc: 97.87%, Sen: 75%, Spec: 98.3%, Prec: 98%	Physiological ECG data
Nolte et al.[66]	2025	Journal of Sports Sciences	Germany	Orthopaedics	Mixed: Patients with/without ACL injury	549	22.2 (male), 23.0 (female)	67	Predictive & Estimation models	Prediction of participants being ACL-injured or not	RF	AUC: 0.90 (male), AUC: 0.92 (female)	Isokinetic strength test data
Nonnenmacher et al.[67]	2025	Bone & Joint Open	Germany	Orthopaedics	Athletes with periacetabular osteotomy	235	31.9	17	Predictive & Estimation models	Prediction of early RTS at 3 and 6 months after surgery	LR, Conditional inference tree	Early RTS associated with surgical approach, sport frequency, psychological factors, and pain; delayed RTS with male sex and older age.	Preoperative demographic and patient-reported questionnaire data
Nose-Ogura et al.[68]	2025	Physician and Sportsmedicine	Japan	Endocrinology	Athletes	614	20.9 (development), 19.6 (validation)	0	Predictive & Estimation models	Prediction of low bone mineral density	LASSO	Development AUC 0.89; Validation AUC 0.74; Sensitivity 0.83; NPV 0.85	Preoperative questionnaire and dual-energy X-ray absorptiometry data
Ohlsen et al.[69]	2025	Cureus	USA	Orthopaedics	NA	NA	NA	NA	Reasoning & Summarization assistance models	Agreement of LLM recommendations with clinical guidelines for ACL and meniscal injuries	ChatGPT-4o, Gemini 2.5 Pro	ChatGPT: 82% agreement, Gemini: 73% agreement; no significant difference between models	Text-based conversation data
Oliver et al.[70]	2020	Journal of Science & Medicine in Sport	England	Orthopaedics	Youth elite football	355	14.3	100	Predictive & Estimation models	Prediction of non-contact lower extremity injury	Multivariate LR, supervised learning DT	DT: AUC: 0.663, Sen: 55.6%, Spec: 74.2%	Preseason neuromuscular screening data and anthropometric measures
Ozbek et al.[71]	2025	Arthroscopy	Turkey / USA	Orthopaedics	NA	NA	NA	NA	Reasoning & Summarization assistance models	Quality assessment of LLM responses to hip arthroscopy patient questions	ChatGPT 4.0	20/25 rated “excellent”; 5/25 “satisfactory”	Text-based conversation data
Pérez-Contreras et al.[72]	2025	Applied Sciences-Basel	Chile	Orthopaedics	Professional football	41	22.3	56	Predictive & Estimation models	Prediction of non-contact muscle injury risk	LR, DT, KNN, RF, GBoost, NN	KNN: Acc 87%, AUC 0.87; Gradient Boosting: Acc 84%, AUC 0.90; Logistic Regression AUC 0.50	Preseason biomechanical and training load data
Piłka et al.[73]	2023	Sensors	Poland	Orthopaedics	Football	36	24	100	Predictive & Estimation models	Prediction of football injury	XGBoost	Prec: 92.4%, recall: 96.5%, F1: 94.4%	Training and match load data
Quinn et al.[74]	2024	Arthroscopy	USA	Orthopaedics	NA	NA	NA	NA	Reasoning & summarization assistance	LLM to test quality of information with regard to ACL reconstruction	ChatGPT-4, Gemini	ChatGPT-4 and Gemini: Overall good ability to generate accurate and relevant responses	Text-based conversation data
Rossi et al.[75]	2023	Sport Sciences for Health	Italy	Orthopaedics	Elite football	18	24.7	100	Predictive & Estimation models	Prediction of non-contact injury risk	DT, GBoost, k-mean cluster	Acc increased to 63% (15% improvement) after blood profile was added to workload-only models	GPS-derived external workload and blood biomarker data
Richter et al.[76]	2023	Sports Biomechanics	Norway	Orthopaedics	Elite football and handball	822	21	0	Predictive & Estimation models	Prediction of participants with previous-/future-/no ACL injury	DT, RF, discriminant analysis, NB, KNN, SVM, LR, NN	Cluster of models: Average AUC 0.62, Sen: 0.59, Spec: 0.58	Biomechanical data
Robinson et al.[77]	2022	American Journal of Physical Medicine & Rehabilitation	USA	Neurology	Athletes	273	21	52	Predictive & Estimation models	Prediction of prolonged recovery after SRC	DT	Acc: 0.7636, Sen: 0.6429, Spec: 0.8889, PPV: 0.8571, NPV: 0.7059	Symptom evaluation data (SCAT5)
Rommers et al.[78]	2020	Medicine & Science in Sports & Exercise	Belgium	Orthopaedics	Youth elite football	734	11.7	100	Predictive & Estimation models	Prediction of musculoskeletal injury	XGBoost	Acc: 85%, Prec: 85%, recall: 85%	Preseason anthropometric, motor coordination and physical performance data
Ruddy et al.[79]	2018	Medicine & Science in Sports & Exercise	Australia	Orthopaedics	Australian football	362	23.2 (2103 season), 25.0 (2015 season)	10	Predictive & Estimation models	Prediction of hamstring injury	NB, LR, RF, SVM, NN	Median of all 5 models: AUC: 0.58 (2013 season), 0.57 (2015 season)	Preseason demographic, injury history and strength test data
Ruiz-Pérez et al.[80]	2021	Frontiers in Psychology	Spain	Orthopaedics	Elite Futsal	139	22.5	52	Predictive & Estimation models	Prediction of soft tissue injury	C4.5, Alternating DT, SVM with SMO, KNN, Instance-Based Learning	Various models: AUC: 0.701 to 0.767	Preseason psychological and neuromuscular data
Saghafi et al.[81]	2018	Proceedings of SPIE	USA	Neurology	Youth and high-school football	122	9 to 18	N/A	Classification & Pattern recognition	Classification of white matter changes after head impact exposure	CNN	AUC: 85.71%, F1: 83.33%	Neuroimaging and biomechanical data
Saglam et al.[82]	2025	BMC Medical Informatics & Decision Making	Turkey	Orthopaedics	NA	NA	NA	NA	Reasoning & Summarization assistance models	Comparison of GPT’s in clinical decision-making	GPT-4, GPT-3.5	GPT-4 significantly outperformed GPT-3.5 (*p* < 0.001; Cohen’s d = 1.42); higher treatment and rehabilitation suitability (*p* < 0.001)	Text-based conversation data
Schulc et al.[83]	2024	Orthopaedic Journal of Sports Medicine	USA	Orthopaedics	Professional athletes with ACL injury	129	NR	NR	Diagnostic & Detection models	Identification of ACL injury through video analysis	Recurrent NN	AUC: 0.88, F1: 0.63	Video-derived biomechanical data
Shibata et al.[84]	2019	Journal of Orthopaedic Science	Japan	Orthopaedics	Patients with ACL-R	386	23.7	53	Predictive & Estimation models	Prediction of quadriceps strength recovery 6 months after ACL-R	DT, Stepwise multiple linear regression	Preoperative QSI, age, and pre-injury Tegner score predicted 6-month QSI; decision tree correctly classified 46.8% of cases	Preoperative isokinetic quadriceps strength, demographic, clinical and intraoperative finding data
Song et al.[85]	2022	Wireless Communications & Mobile Computing	China	Orthopaedics	Track and field	12	19.5	N/A	Predictive & Estimation models	Evaluation of rehabilitation effectiveness using AI and virtual reality-assisted training	Probabilistic NN, SVM	AI+virtual reality group achieved > 96% physical function recovery; overall rehabilitation score 93.79 vs 82.38 (control)	Physiological blood measures, functional, strength and speed assessment data
Sparks et al.[86]	2024	JB & JS Open Access	USA	Orthopaedics	NA	NA	NA	NA	Reasoning & summarization assistance	LLM to investigate accuracy of patient education with regard to orthopaedic conditions	ChatGPT-3.5	Moderately accurate outputs for general inquiries. Lack in the quantity of information for risk factors and treatment options.	Text-based conversation data
Stirling et al.[87]	2025	Journal of Orthopaedic Research	Canada	Radiology	Patients with ACL injury	100	33.6	32	Classification & Pattern recognition	Automated quantification of bone marrow lesion volume and association with pain outcomes	CNN	Bone marrow lesions present in 95%; 96.1% volume reduction at 1 year (*p* < 0.001); baseline BML volume modestly associated with symptoms	MRI and patient-reported questionnaire data
Tamez-Peña et al.[88]	2021	Frontiers in Neurology	Unknown	Neurology	Student athletes	122	18.8	53	Classification & Pattern recognition	Classification of SRC	SVM	Sen: 0.80, Spec: 0.74	Neuroimaging radiomic data
Tedesco et al.[89]	2020	Sensors	Ireland	Orthopaedics	Non-elite rugby	12	26	100	Diagnostic & Detection models	Identification of gait patterns in participants with or without ACL injury	KNN, NB, SVM, GBoost, MLP, stacking	MLP: Acc: 73.07; GBoost: Sen: 81.8%	Inertial sensor data
Thanjavur et al.[90]	2021	Frontiers in Human Neuroscience	Canada	Neurology	Adolescent athletes	58	13.4 (injured), 14.7 (uninjured)	100	Classification & Pattern recognition	Classification of SRC	ConcNet 2 and 3	Acc: 94%, AUC: 0.971	EEG data
Tsilimigkras et al.[91]	2024	Journal of Sports Science & Medicine	Greece	Orthopaedics	Professional football	25	N/A	100	Predictive & Estimation models	Prediction of muscle injury risk	SVM	Acc: 0.78, Sen: 0.73, Spec: 0.85	Physiological and mechanical workload data
Usami et al.[92]	2024	Knee Surgery, Sports Traumatology, Arthroscopy	Japan	Orthopaedics	Mixed: Patients with ACL-R	386	25.1	49	Diagnostic & Detection models	Detection of graft rupture and contralateral ACL injury	NN	AUC: 0.81 (graft rupture), 0.74 (contralateral ACL injury)	Clinical, demographic, and surgical medical record data
Vallance et al.[93]	2020	Applied Sciences-Basel	France	Orthopaedics	Elite football	40	29.4	100	Predictive & Estimation models	Prediction of non-contact injury risk	KNN, DT, RF, XGBoost, SVM, MLP, Linear discriminant analysis, LR, Ridge regression, NB	1-month prediction: XGBoost AUC 0.97; 1-week prediction: questionnaires outperformed GPS data; internal load strongest short-term predictor	GPS-derived external load, rating of perceived exertion and well-being questionnaire data
Valle et al.[94]	2022	Sports Medicine	Spain	Orthopaedics	Elite football	76	24.2	100	Predictive & Estimation models	Prediction of recovery	Linear regression, RF, XGBoost	XGBoost (days to recovery): Mean absolute error: 9.78884, Root mean squared error: 12.1450, R-squared: 0.4847	Clinical and MRI data
Villarreal-Espinosa et al.[95]	2024	Knee	USA	Orthopaedics	NA	NA	NA	NA	Reasoning & summarization assistance	LLM for patient education with regard to ACL surgery	ChatGPT-4	5/10 responses completely accurate (by two reviewers), and 3/10 completely accurate (by at least one reviewer). Inter-rater reliability: weighted kappa: 0.57. 80% of responses were reproducible over time	Text-based conversation data
Wang et al.[96]	2026	Scientific Reports	Various	Orthopaedics	Professional football	312	24.7	100	Predictive & Estimation models	Prediction of non-contact lower extremity injuries	RF, SVM, GBoost, DNN, Ensemble model	Ensemble AUC 0.759	Isokinetic strength, training load, injury history and biomechanical data
Weng et al.[97]	2025	Journal of Sports Sciences	Taiwan	Orthopaedics	Various level baseball	98	18.0 (injured), 17.5 (uninjured)	100	Predictive & Estimation models	Prediction of upper extremity injury	GIRD, LR, RF, CatBoost, SVM	CatBoost: AUC: 0.66, Acc: 0.70	Clinical and musculoskeletal data
Yates et al.[98]	2025	BMJ Open Sport & Exercise Medicine	England	Neurology	Contact sport athletes	375	24.2	78	Predictive & Estimation models	Prediction of SRC recovery	RF	Acc: 94.6%, Sen: 100%, Spec: 93.8%, PPV: 71.4%, NPV: 96.3%	Clinical and MRI data
Ye et al.[99]	2023	Frontiers in Physiology	Netherlands	Orthopaedics	Elite runners	64	N/A	65	Predictive & Estimation models	Prediction of running injury	GASF-DCAE-DNN	AUC 0.985, Gmean: 0.930, Sen: 0.997, Spec: 0.868. Test: AUC: 0.891, Gmean: 0.830, Sen: 0.816, Spec: 0.845	Longitudinal training load, and physiological performance data
Yüce et al.[100]	2024	Cureus	Turkey	Orthopaedics	NA	NA	NA	NA	Reasoning & summarization assistance	LLM for patient education with regard to sports surgery	ChatGPT-4	DISCERN: 44.75 points. Sports surgery-specific scoring: 13.3 points	Text-based conversation data
Zhan et al.[101]	2025	Arthroscopy	China	Orthopaedics	Mixed: Patients with MPFL-R	218	NR	NR	Predictive & Estimation models	Prediction of clinical outcomes in patients with medial patello-femoral ligament reconstruction	RF, LR, SVM, DT, implemented MLP, KNN	Various models: AUC: 0.760 to 0.969, and Acc: 76.8% to 95.2% (Subjective outcomes); AUC: 0.952, and Acc. 95.2% (Return to pre-injury sport); AUC: 0.756, and Acc: 75.4% (Return to pivoting sports); AUC: 0.943, and Acc: 94.9 (Recurrent instability)	Clinical, demographic and radiographic data
Zhan et al.[102]	2023	Journal of Sport & Health Science	USA	Neurology	Mixed: Lab, MMA, American football, automobile, NASCAR	3262	NR	NR	Classification & Pattern recognition	Classification of head impact subtypes	RF	Acc: 96%	Biomechanical data from head impact recordings
Zhang et al.[103]	2022	Contrast Media & Molecular Imaging	China	Radiology	Mixed: Patients with ACL injury	90	39	60	Diagnostic & Detection models	Image optimization to assess ACL integrity	iDose4 Iterative Reconstruction Algorithm.	Improved image quality	Clinical CT data
Zhu et al.[104]	2026	BMC Sports Science, Medicine and Rehabilitation	China	Orthopaedics	Patients with ACL-R	30	31.9 (RTS group), 36.9 (no-RTS group)	73 (RTS group), 60 (no RTS group)	Classification & Pattern recognition	Identification of urinary proteomic biomarkers associated with RTS	LASSO	AUC range 0.827–0.876	Urinary proteomic, isokinetic strength, hop test, thigh circumference and patient-reported questionnaire data
Zhu et al.[105]	2026	Journal of Clinical Medicine	China	Orthopaedics	Patients with ACL-R	79	30.9	89	Predictive & Estimation models	Evaluate effectiveness of a rehabilitation protocol incorporating an AI-based assessment and correction system on functional recovery	Intelligent Functional Movement and Physical Fitness Assessment System (ZD-200S-JG)	Trial group showed significantly greater improvements in patient-reported outcomes and range of motion and rehabilitation adherence	Non-wearable three-dimensional motion capture, clinical and patient-reported questionnaire data

ACL-RSI = Anterior cruciate ligament-return to sport after injury, Acc = Accuracy, AdaBoost = Adaptive boosting, AI = Artificial intelligence, AUC = Area under the receiver operating curve, CART = Regression tree analysis, CatBoost = Categorical boosting, CNN = convolutional neural networks, DT = Decision tree, CT = Computer tomography, ECG = electrocardiogram, EEG = electrocochleography, EMO = Electromyography, ENPLR = Elastic-net penalized logistic regression, F1 = F1-Score, F2 = F2-Score, GASF-DCAE-DNN = Gramian Angular Summation Field-Deep Convolutional Auto-Encoder-Deep Neural Network, GBoost = Gradient boosting, GPS = Global positioning system, GPT = Generative pre-trained transformer, IKCD = International Knee Documentation Committee, KNN = K-nearest neighbor, LASSO = Least Absolute Shrinkage and Selection Operator, LE = Lower extremity, LR = Logistic regression, MLP = Multilayer perceptron, MRI = Magnetic resonance imaging, NA = Not applicable, NB = Naïve Bayes, NN = Neural networks, NPV = Negative predictive value, NR = Not reported, OR = Odds ratio, PASS = Patient acceptable symptom state, PPV = Positive predictive value, Prec = Precision, RF = Random forest, RTS = Return to sport, Sen = Sensitivity, Spec = Specificity, SRC = Sports-related concussion, SVM = Support Vector Machine, Youden = Youden index

### AI applications

The AI application categories of the included studies were as follows: predictive and estimation models (*n* = 56, 57.7%), classification and pattern recognition models (*n* = 20, 20.6%), diagnostic and detection models (*n* = 11, 11.3%), and reasoning and summarization assistance models (*n* = 10, 10.3%) (Table 1). Within orthopaedics, predictive models were most common (43/68, 63.2%), whereas classification models were most common within neurology (9/18, 50.0%). Across AI applications, predictive and estimation models were mainly employed to forecast injury risk (*n* = 34), or recovery-related outcomes (*n* = 15), including RTS probability and functional improvement after orthopaedic injury/surgery. Classification and pattern-recognition models (*n* = 17) were primarily used to distinguish between injured and uninjured states, classify SRC or gait patterns, and identify biomechanical risk clusters. Diagnostic and detection models (*n* = 8) were mainly applied for image- or video-based injury identification, such as ACL or lumbar spine pathology. Reasoning and summarization assistance studies (*n* = 10) exclusively investigated LLM models (ChatGPT, Gemini, Bard, DeepSeek) for patient education and information quality assessment.

### Annual distribution and countries of published studies

**Fig. 2:**
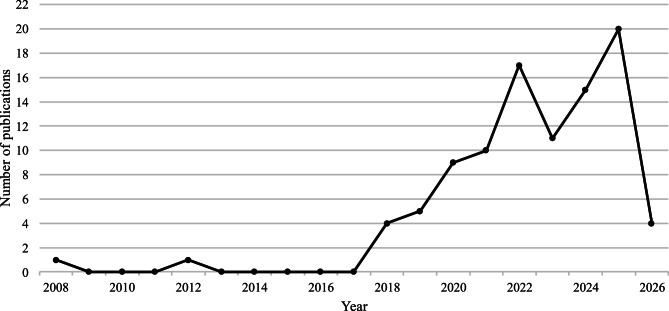
Annual distribution of published studies. For year 2026, only publications up until 5 of February were included

Only two studies were identified before 2018, while the number increased thereafter, and peaked in 2025 (20 publications, 20.6%) (Fig. 2 and Table 1). Of all included studies, 86.6% were published between 2020 and 2026. Of the included studies, most studies were conducted in the USA (*n* = 26, 26.8%), followed by China (*n* = 10, 10.3%), Canada (*n* = 6, 6.2%), Spain (*n* = 5, 5.2%), Japan, Germany, and Turkey (*n* = 4, 4.1%, respectively), Chile, France, and Netherlands (*n* = 3, 3.1%, respectively), Italy, South Korea, England, United Kingdom, and Iran (*n* = 2, 2.1%, respectively), and Australia, Ireland, Norway, Poland, Finland, Iceland, Croatia, Sweden, Romania, Belgium, Portugal, Taiwan, and Greece each had one (1.0%) contribution, while six (6.2%) studies were conducted across multiple countries, and one (1.0%) study had unknown origin.

### Fields of population

**Fig. 3:**
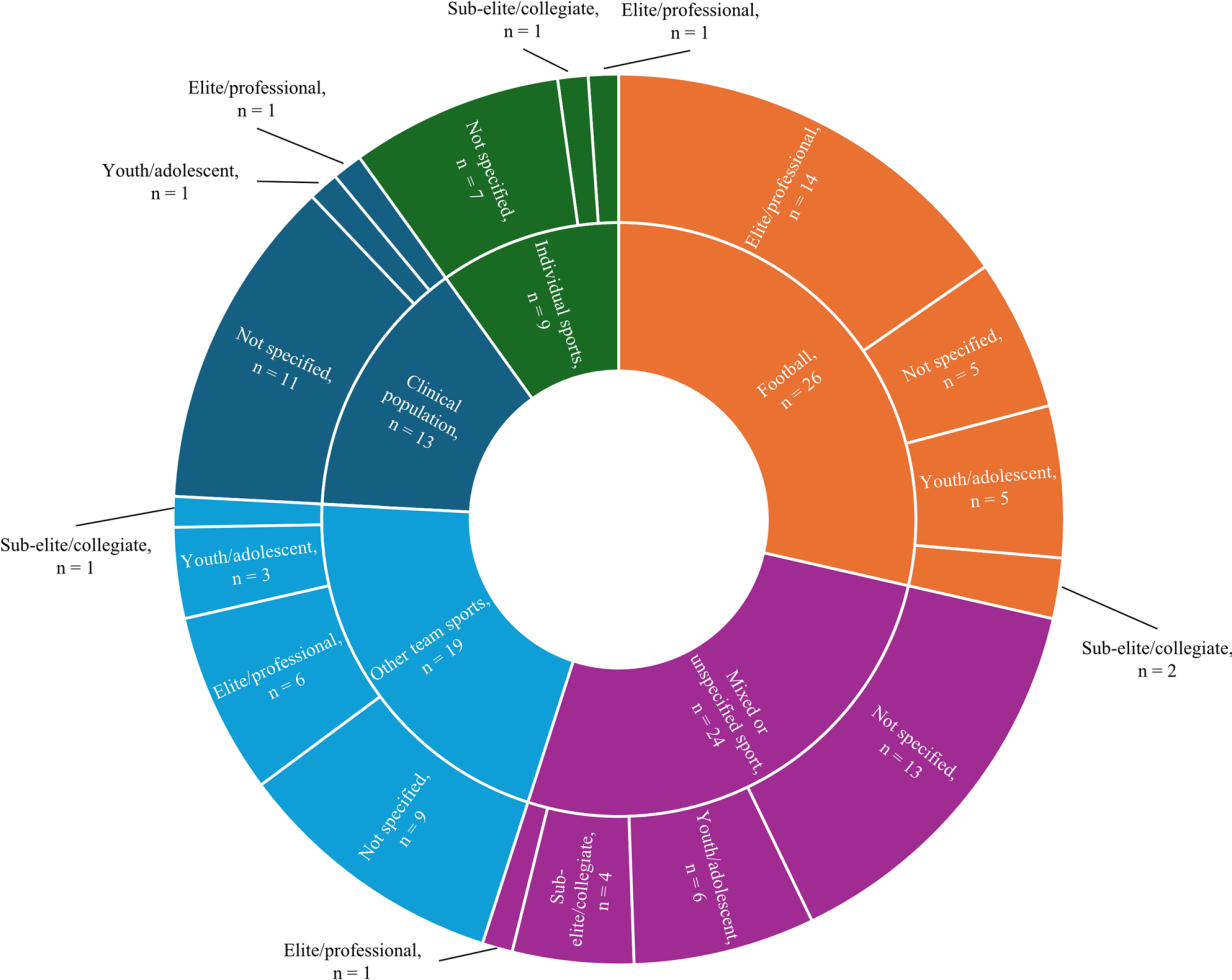
Distribution of study populations across included studies. The inner ring represents sport, and the outer ring level of participation. *n* = number

The most common specific fields of population were football (*n* = 26), followed by basketball (*n* = 6), rugby (*n* = 4), handball (*n* = 4), and runner-related populations (*n* = 4). Studies which included mixed athlete populations (*n* = 22) and a combination of athletes and non-athletes (*n* = 10) were also common. The distribution of study population across sport categories and levels of participation is presented in Fig. 3. Data environments differed by sport: football and running studies predominantly analyzed global positioning system (GPS)-based workload and accelerometry, whereas basketball, handball, and volleyball more often used laboratory biomechanical tests, balance/fatigue assessments, or physical performance analysis. Rugby studies included video-derived tackle characteristics. Sample sizes ranged from small elite squads (*n* = 10) to large youth/registry cohorts (*n* = 24,561), with prospective designs typical for load-monitoring studies and cross-sectional designs were common for imaging/electroencephalogram (EEG) tasks. Across sports, participant sex distribution varied across studies, and no studies specifically included para-sport populations. While many studies integrated multidimensional inputs that combined load, wellness/psychological, and clinical variables, relatively few integrated truly multimodal data that combined different modalities such as imaging, senosor-based signals, or video [15, 26, 40, 44, 49, 68, 75, 85, 87, 101, 104, 105].

### AI models

The five most included models used overall were random forest (RF) (*n* = 29), support vector machine (SVM) (*n* = 27), decision tree (DT) (*n* = 20), neural network (NN) (*n* = 15), and XGBoost (*n* = 12) (Table 2). Among the three most common population groups, RF models were most frequently used in studies which involved general athlete populations (9/20, 45.0%) and mixed populations (4/7, 57.1%). In studies focused on football, SVM and XGBoost models were the most commonly used, each applied in 10 out of 25 studies (40.0%). Beyond these core algorithms, categorical boosting (CatBoost), adaptive boosting (AdaBoost), Light gradient boosting machine, and elastic-net penalized regression were frequent ensemble or regularized variations, and 14 studies explored newer architectures such as deep-learning models (TabNet, ConcNet, CNNs, feedforward NNs, recurrent NNs [RNNs], Faster region-based CNN [Faster R-CNN]) for imaging or signal-based classification. Model selection generally reflected data type: tree-based ensembles and SVMs dominated structured tabular datasets (e.g., sensor, GPS, clinical, or performance data), while CNNs and RNNs were used for imaging and video, and task-specific networks such as ConcNet for EEG. Unsupervised clustering (umap, k-means, subgroup discovery) appeared occasionally for identifying biomechanical or risk profiles. In total, 42 studies incorporated explainable AI methods (feature importance or Shapley additive explanation values). Ten studies investigated LLMs (ChatGPT, Gemini, Bard, DeepSeek) for patient education rather than prediction. Across studies, reported model performance was generally favorable, with accuracy ranging between 63 and 100%, area under the receiver operating characteristics curve (AUC) between 0.57–0.985, sensitivity between 35 and 100%, precision between 63.6%-100%, and F1-score between 0.6–0.944 depending on study design and outcome definition; in studies that compared approaches, ML models generally demonstrated higher discriminative performance than traditional regression methods [40, 53, 70, 72]. However, the model performance metrics were predominantly derived from internal cross-validation (*n* = 68), or no validation (*n* = 15) procedures, while external/temporal validation and reporting of calibration metrics were rare (*n* = 4), which underscores that while AI model performance was often strong, methodological maturity and reproducibility remain limited across sports medicine applications.

**Table 2:**
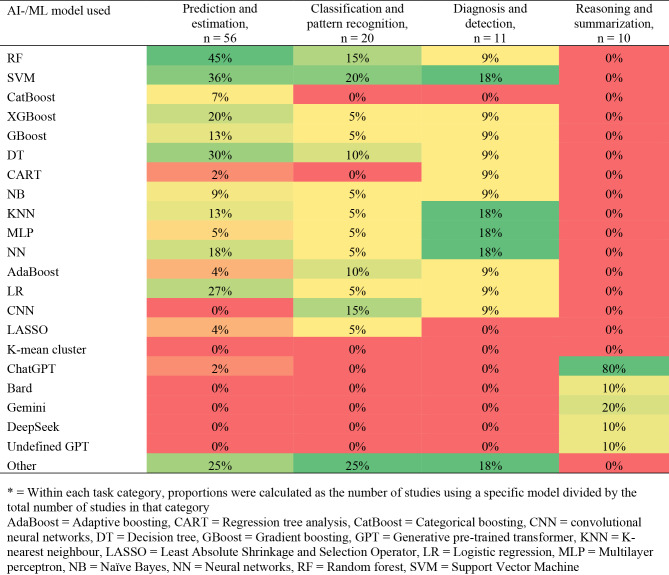
Heatmap of AI-/ML model(s) used in each included study*

### Studies in orthopaedics

Within orthopaedics (*n* = 68), AI was applied to three main areas: injury prediction, diagnostic imaging/video analysis, and outcome estimation after ligament reconstruction (Table 1). Common inputs included isokinetic knee strength, hop and balance tests, jump biomechanics, GPS-derived loads, clinical/radiographic fields, and MRI/computer tomography (CT) or match-video frames. Most studies (43/68, 63,2%) implemented predictive/estimation models, which typically used preseason screening (neuromuscular tests and anthropometrics), training-load/GPS and wellness logs, or clinical/surgical registry data to forecast ACL injury/reinjury, muscle strain, or RTS and functional recovery. Discrimination was generally poor to excellent (AUC 0.61–0.97, accuracy 65–98%), with tree-based ensembles (RF, XGBoost, gradient boosting) frequently achieved AUC ≥ 0.80 [7, 40, 43, 47, 60, 61, 78] and, in a few ACL-specific outcome models, approached 0.90–0.95 [48, 66]. Diagnostic/detection studies (9%) focused on imaging and video, for example, CNN or RNN models to identify ACL injury from online match footage or to distinguish lumbar spondylolysis from non-specific low-back pain, reported accuracies of 73–96%. Beyond imaging, several works modeled post-operative trajectories (e.g., graft rupture risk, subjective function, and psychological readiness), drawing on isokinetic strength, hop tests, y-balance tests and patient reported outcomes. A small subset evaluated LLMs for orthopaedic patient education (e.g., ACL reconstruction and sports surgery information quality), which represented methodological exploration rather than prediction. Taken together, the orthopaedic literature reports strong within-sample performance on structured, tabular data and promising results for image/video-based detection, however, external/temporal validation and diverse cohorts (including female athletes) remain limited, which constrained generalizability.

### Studies in neurology

In neurology-focused research (*n* = 18), nearly all studies (17/18, 94.4%) examined SRC, that used models to predict recovery time or classify concussion presence/severity (Table 1). Inputs were predominantly clinical and neurocognitive assessments (e.g., sport concussion assessment tool 3/5, vestibular ocular motor screening, symptom inventories, prior SRC, and time-to-clinic), sensor-based biomechanics (head-impact kinematics, and dual-task gait/behavioral performance), and physiological signals including EEG (resting-state and task-based) and radiomics from MRI/diffusion tensor imaging, where several studies combined these into multimodal feature sets [15, 26, 32, 49, 81, 98]. Reported performance was moderate to excellent, with AUC 0.70–0.96 and accuracy 75–96%. For recovery-time prediction, decision-tree and boosting approaches achieved AUC ~0.80 and sensitivity > 0.90 for early-recovery classification in pediatric and youth cohorts [6, 22]. For SRC classification, SVM/RF/boosting models commonly reached AUC ≥ 0.80 [32, 49, 62], while deep-learning on EEG (e.g., ConcNet) reported the highest accuracy (94%) and AUC (0.971) [90]. Overall, neurology applications show strong within-sample discrimination across both predictive and classification use-cases and growing interest in multimodal modeling. However, external/temporal validation, standardized feature sets, and explainability remain limited, which constrained generalizability beyond single-site cohorts.

### Model validation and translational stage

Across all included studies, the majority relied on retrospective datasets or observational prospective and internal validation procedures, most commonly cross-validation or train-test splits. Only four studies employed external datasets to assess generalizability, and reporting of calibration metrics was uncommon [15, 68, 76, 96]. Prospective implementation within preventive or decision-making workflows was rare, with only one study attempting a feedback-based interventional approach [23]. Two studies evaluated AI-integrated rehabilitation systems within structured training programs, which included one randomized controlled trial in postoperative ACL rehabilitation [105] and one application which combined AI and virtual reality for athlete rehabilitation training [85].

## Discussion

This scoping review provides a synthesis of AI applications across sports medicine, and demonstrates that, although methodological development has accelerated in recent years, most studies remain in an early developmental stage. Artificial intelligence has been widely applied for injury prediction, diagnostic imaging, and recovery estimation across diverse athletic and clinical populations, mostly within orthopaedics and neurology. Despite frequently high reported performance metrics, the literature is characterized by substantial heterogeneity in model selection, data modalities, outcome definitions, and validation procedures. Most studies relied on retrospective or observational prospective datasets and internal validation methods, whereas external or temporal validation and prospective prevention or intervention frameworks were rare. Consequently, the current evidence base does not yet support routine clinical integration of AI-driven decision tools in sports medicine. In practice, AI models should currently be interpreted as adjunct tools to support, rather than replace, clinician judgement in injury risk assessment, diagnosis, and rehabilitation planning.

Although many models demonstrated strong discriminative performance, which often achieved AUC values ≥ 0.80, these findings must be interpreted in the context of important methodological limitations. High within-sample accuracy suggests that AI can effectively identify patterns associated with injury risk, recovery trajectories, or RTS potential. However, the vast majority of studies performed validation within the same dataset, typically through internal cross-validation or train-test splits. Only four studies employed external datasets to assess generalizability [15, 68, 76, 96]. This represents a critical methodological limitation, as internal validation tends to overestimate model performance and fails to account for differences in population characteristics, data collection methods, or sporting environments [106]. In addition, the prediction timeframes across included studies varied, with short-term models typically based on repeated within-subject measurements (e.g., training load or wellness data) and longer-term models relying on baseline variables, which introduces heterogeneity in model design and clinical applicability. Without robust external or temporal validation, the true predictive value and clinical reliability of these models remain uncertain. Accordingly, the literature reflects predominantly early-stage model development, with limited progression toward external validation, prospective integration, or demonstrated impact on clinical decision-making. Future studies should prioritize multi-center external validation across teams, seasons, and demographic groups, to evaluate whether reported performance translates into meaningful clinical utility. Clinically, the uncertainty of findings limits the use of AI models for individual-level decision-making, such as identification of athletes at high risk of reinjury or determination of readiness for RTS, where reliable generalization across populations is important.

While AI-driven prediction models have been reported with strong retrospective accuracy, only Dandriex et al. [23] attempted to integrate predictions into a prospective, feedback-based prevention strategy, in which daily individualized feedback was provided to track-and-field athletes based on self-reported wellness data. However, adherence was low (average daily response rate of 37%), and no significant association with injury burden was observed, although a modest protective effect was suggested among participants with at least 9% response rate [23]. In addition to predictive frameworks, a small number of studies have integrated AI directly into structured rehabilitation programs. For example, one randomized controlled trial evaluated an AI-based assessment and correction system after ACL reconstruction [105], which demonstrated improvements in functional outcomes and rehabilitation adherence, while another study applied AI and virtual reality technology within athlete rehabilitation training [85]. These findings illustrate both the potential and the current limitations of AI-supported frameworks, namely, information alone does not yield benefit unless it is coupled with consistent athlete engagement, integration into clinical or training workflows, and actionable feedback mechanisms capable of influencing real-word decision-making, such as modification of training load, or informing RTS clearance.

Sports injuries are inherently multifactorial, which arise from complex interactions between training load, biomechanics, physical fitness, psychological status, and contextual factors such as playing surface and competition demands [107, 108]. Consequently, models that rely solely on single wellness or workload metrics are unlikely to capture the full spectrum of injury risk. This highlights the need for multimodal data integration that combines these factors to better reflect the complexity of athletic performance and health [109]. Recent work demonstrates that combination of factors such as genetics, biomechanics, nutrition, and training load can improve injury prediction, although the approach was represented within a single tabular data modality rather than true multimodal frameworks [110]. In the context of sports medicine, models intended to inform RTS decisions or reinjury risk estimation must also be interpretable, to allow stakeholders to understand which variables drive predictions and how they align with established clinical reasoning [109], particularly in clinical settings where practitioners must justify decisions related to rehabilitation progression or RTS clearance based on transparent and clinically interpretable information.

The growing use of high-performing yet opaque black-box models, such as DNNs, poses a barrier to practical implementation, as limited explainability can erode user confidence and hinder clinical decision-making [111]. Accordingly, development of AI frameworks that balance predictive strength with transparency will be crucial to support actionable and trustworthy decision support. Future research should extend beyond prediction to design and evaluate controlled, prospective, decision-driven systems that integrate real-time recommendations and test whether AI-supported interventions can meaningfully reduce injury incidence or improve recovery outcomes [112].

Overall, studies that evaluate the capabilities of LLMs in sports medicine suggest that these tools can generate generally accurate and informative responses, particularly for patient education. However, two key concerns were identified. Firstly, Sparks et al. [86] reported that LLM outputs often lacked sufficient detail with regard to risk factors and treatment options. Secondly, Villarreal-Espinosa et al. [95] found that two of ten responses about ACL surgery did not reach a very accurate rating, and that 80% responses were reproducible over time. In addition, LLMs are inherently susceptible to hallucinations, distribution shifts between training data and real-world use, and potential instability of output, all of which may further undermine their reliability in clinical athletic contexts [113–115]. These findings highlight the potential risk that patients may receive incomplete, inaccurate, or inconsistent information. Taken together, LLMs have serve as accessible adjunct educational tools, but current evidence does not support unsupervised clinical deployment. At present, their use should remain supervised by clinicians, particularly when addressing diagnosis, surgical decision-making, or RTS guidance. Future research should focus on benchmarking LLM performance against verified clinical standards and explore how these tools can be safely embedded into patient communication and rehabilitation pathways without compromising information accuracy. Future work should explore fine-tuning and retrieval-augmented generation to incorporate domain-specific knowledge and possibly improve clinical utility and reliability.

Image and diagnostic AI applications showed promise in accuracy for identification of ligament injuries, bone fractures, and concussion-related imaging patterns. These approaches leverage automated feature extraction from radiological or video data, which reduces reliance on manual interpretation [116]. However, most studies were limited by small sample size, and lack of external or multi-center validation, thus restricting clinical applicability [117]. Furthermore, image-based models often function as black boxes, with limited explainability of which image features drive decisions, which underscores the need for interpretable visualization methods to support clinical implementation. In practice, AI applications may assist clinicians in improving diagnostic accuracy and reducing inter-observer variability, although current limitations restrict independent use in clinical decision-making.

Across studies in which ML models were used alongside traditional regression approaches [40, 53, 70, 72], ML algorithms demonstrated higher discriminative performance. However, these studies were limited to within-sample or internally validated analyses, and formal benchmarking with strict comparisons between methods was rare. Consequently, although ML models may offer incremental gains in discrimination, it remains unclear whether these improvements translate into superior calibration, generalizability, or meaningful enhancement of clinical decision-making. Future research should therefore include structured comparative evaluations that assess not only discrimination but also calibration, interpretability, and net clinical benefit to determine whether increased algorithmic complexity provides practical advantages over established biostatistical methods.

Across studies, substantial model heterogeneity was observed, with each investigation employing diverse algorithms, feature sets, and outcome definitions. This diversity underscores the exploratory nature and limits consensus on optimal model families or architectures for specific sports or data types [118]. In parallel, the generalizability of existing models remains uncertain, as most datasets were small, and single-site, with minimal or no inclusion of youth, or para-sport athletes and limited representation outside Europe and North American leagues [106]. These sampling biases restrict the broader applicability of reported findings. Furthermore, the validity of any AI model ultimately depends on the quality of data input [119]. Inconsistent data collection methods, and missing contextual variables can all undermine predictive accuracy, regardless of algorithmic sophistication [119]. Collectively, this methodological variability reinforces that the field remains in developmental phase, in which foundational issues of data standardization, and reporting transparency must be addressed before reliable large-scale implementation can be achieved. Addressing these foundational issues through standardized data handling, and transparent reporting will be important to build robust, and trustworthy AI systems in sports medicine.

This review indicates that most current applications of AI and ML in sports medicine remain in an early developmental exploratory stage and should be interpreted cautiously in clinical settings. Consequently, these models should not yet be relied upon to independently guide clinical decision‑making, diagnosis, prognosis, or RTS recommendations. Instead, the current role of AI and ML models lies in supporting clinicians by synthesizing complex data patterns that may not be readily identifiable through traditional assessment alone. At present, many models rely on retrospective or prospective observational datasets, lack external validation, and have not undergone evaluation in real‑world clinical workflows, which limits their immediate clinical reliability and generalizability. Furthermore, the majority of the included studies focused on orthopaedics or neurology, therefore, the findings observed in these areas may not be directly applicable to other domains within sports medicine. The main contribution of this review was to provide clinicians and researchers with a clearer understanding of the current maturity and limitations of AI and ML technologies within the field. By synthesizing the available evidence, this work aims to support more informed, critical appraisal of algorithm‑generated outputs and to help clinicians recognize when such tools may complement rather than replace clinical reasoning. Furthermore, the review outlines priority areas in which rigorous model validation, prospective study designs, bias assessment, and implementation research are important. Addressing these gaps will be crucial to ensure that future AI‑ and ML‑based tools can transition safely and effectively from experimental settings to practical, patient‑centered clinical application.

This study has several limitations. The included studies varied widely in sport, level of play, sample size, data modality, outcomes, and performance metrics which did not allow for a quantitative synthesis and limits comparison across models. Therefore, it was determined to synthesize narratively to emphasize patterns rather than pool effects. In addition, due to the heterogeneity of included studies, no risk of bias or quality assessment was performed. Moreover, the predominance of internal validation limits the generalizability of findings. Thus, results must be interpreted cautiously. Furthermore, publication bias might skew the picture of the accurate results of included studies, and true performance of AI models might not be captured. Given the rapid growth of this field, relevant studies may have been published after our search and were therefore not captured. Engineering-led studies focused solely on algorithmic development without application to human data in a sports medicine context were excluded; therefore, the technical foundation of AI methods may be underrepresented. To organize this heterogeneous literature, AI applications were pragmatically grouped into four mutually exclusive categories. While this approach facilitates synthesis, it simplifies the inherently multidimensional nature of AI systems, which often vary substantially in data sources, modelling approaches, and validation strategies within the same clinical task. Accordingly, the categories should be interpreted as heuristic groupings to support interpretation rather than rigid distinctions between fundamentally different AI methodologies. Given the large number of included studies, it was not feasible to verify the reproducibility of individual models or reported performance metrics; limited availability of code, data, and methodological detail further restricted independent validation and highlights the need for improved transparency in AI- and ML-based sports medicine research.

## Conclusion

The use of AI applications in sports medicine demonstrate strong within-sample discriminative performance for injury risk, recovery, and diagnostic imaging, yet most remain limited to retrospective analysis with limited external validation and minimal evidence of clinical workflow integration. This review shows that the field is characterized by substantial methodological heterogeneity and limited progression toward prospective implementation. For clinicians within sports medicine, current AI tools should therefore be regarded as exploratory decision-support adjuncts rather than implementation-ready systems.

## Electronic supplementary material

Below is the link to the electronic supplementary material.

Supplementary Material 1

## Data Availability

This scoping review is based on previously published studies. The data-charting spreadsheet generated during the review is available from the corresponding author on reasonable request.
