# Supplementary material for: Artificial intelligence and machine learning in sports medicine: mapping clinical tasks and assessing clinical maturity - a scoping review
Source: BMC Med Inform Decis Mak. 2026 Jun 12;26:212. doi: 10.1186/s12911-026-03615-w (PMC13263937; doi:10.1186/s12911-026-03615-w)
Supplement: Supplementary file 1 — Supplementary Material 1 [file 12911_2026_3615_MOESM1_ESM.docx]

**Supplementary information**

| **#** | **Searches** | **Results** |
| --- | --- | --- |
| 1 | exp Sports Medicine/ | 11996 |
| 2 | Athletic Injuries/ | 33769 |
| 3 | exp Physical Therapy Modalities/ and exp Sports/ | 19504 |
| 4 | ((sport* or athletic*) and (biomechanic* or injur* or medicin* or performance* or physiolog* or recover*)).ab,kf,ti. | 85290 |
| 5 | ((sport* or athletic*) and (physiotherap* or neurophysiotherap* or physical therap* or rehab* or neurorehab*)).ab,kf,ti. | 14496 |
| 6 | 1 or 2 or 3 or 4 or 5 | 130120 |
| 7 | exp Artificial Intelligence/ | 269383 |
| 8 | ((artificial or machin* or comput* or automated) adj3 (intellig* or superintellig* or reason* or vision* or knowledge* or inferen*)).ab,kf,ti. | 122420 |
| 9 | (knowledge adj3 acquisition*).ab,kf,ti. | 5295 |
| 10 | (AI-guid* or AI-assist* or AI-aid* or AI-augment* or AI-base* or AI-system* or AI-technolog* or AI-power* or AI-process*).ab,kf,ti. | 21076 |
| 11 | ((comput* or algorithm*) adj3 heuristic*).ab,kf,ti. | 2291 |
| 12 | (hyperheuristic* or hyper-heuristic* or metaheuristic* or meta-heuristic*).ab,kf,ti. | 2883 |
| 13 | ((multicriteria or multi-criteria or "multiple criteria" or multiobjective or multi-objective or multiattribute or multi-attribute) adj3 (decision* or optimi* or algorithm*)).ab,kf,ti. | 9068 |
| 14 | MOEA.ab,kf,ti. | 267 |
| 15 | (analytic* adj3 hierarch*).ab,kf,ti. | 3233 |
| 16 | (cognitive adj3 (comput* or robotic* or technol*)).ab,kf,ti. | 4856 |
| 17 | ((machine or transfer or deep or hierarchical or labeled data or supervised or semisupervised or unsupervised or network* or Bayesian or manifold*) adj3 learn*).ab,kf,ti. | 287858 |
| 18 | ((automat* or software or system* or technol* or pattern* or optical character*) adj3 recognit*).ab,kf,ti. | 39839 |
| 19 | (back* adj3 propagation*).ab,kf,ti. | 4177 |
| 20 | "backpropagation*".ab,kf,ti. | 2982 |
| 21 | ((classificat* or detect* or learn*) adj3 algorithm*).ab,kf,ti. | 69284 |
| 22 | "classifier*".ab,kf,ti. | 53408 |
| 23 | ((confusion or error or matching) adj3 matri*).ab,kf,ti. | 2955 |
| 24 | ((feature or edge) adj3 (detect* or extract* or learn* or rank* or select*)).ab,kf,ti. | 57887 |
| 25 | (vector* adj3 (machine* or support or classif* or network* or regression*)).ab,kf,ti. | 43115 |
| 26 | (expert adj3 (system or systems)).ab,kf,ti. | 4658 |
| 27 | (fuzz* adj3 (logic* or system*)).ab,kf,ti. | 6089 |
| 28 | (hidden Markov or Markov state model*).ab,kf,ti. | 6701 |
| 29 | "iterative closest point*".ab,kf,ti. | 679 |
| 30 | (knearest neighbo* or k nearest neighbo*).ab,kf,ti. | 9650 |
| 31 | "kernel*".ab,kf,ti. | 33487 |
| 32 | (temporal difference adj3 (algorithm* or learn*)).ab,kf,ti. | 223 |
| 33 | (qlearn* or q-learn*).ab,kf,ti. | 852 |
| 34 | ((multifactor* or multi-factor*) adj3 dimension* adj3 reduction*).ab,kf,ti. | 1472 |
| 35 | (online analytic* adj3 process*).ab,kf,ti. | 63 |
| 36 | OLAP.ab,kf,ti. | 89 |
| 37 | ((outlier or anomaly) adj3 detect*).ab,kf,ti. | 5362 |
| 38 | (radial bas* adj3 function*).ab,kf,ti. | 3517 |
| 39 | (general* adj3 regression adj3 network*).ab,kf,ti. | 417 |
| 40 | "random forest*".ab,kf,ti. | 43469 |
| 41 | (knowledge base or knowledge bases).ab,kf,ti. | 12196 |
| 42 | ((biologic or biological or biomedical or gene) adj3 ontolog*).ab,kf,ti. | 42835 |
| 43 | (natural language adj3 (process* or system*)).ab,kf,ti. | 14401 |
| 44 | (language process* or NLP).ab,kf,ti. | 21514 |
| 45 | ((neural or deep or convolutional or feedforward or feed-forward) adj3 network*).ab,kf,ti. | 157883 |
| 46 | "memristor*".ab,kf,ti. | 2896 |
| 47 | "molecular dock*".ab,kf,ti. | 72795 |
| 48 | "perceptron*".ab,kf,ti. | 7759 |
| 49 | (connect* adj3 (model* or network* or system*)).ab,kf,ti. | 39414 |
| 50 | (autoencoder* or auto-encoder*).ab,kf,ti. | 7032 |
| 51 | ((companion or social or socially) adj3 (robot or robots)).ab,kf,ti. | 1417 |
| 52 | (soft adj3 (robotic* or telerobotic*)).ab,kf,ti. | 3804 |
| 53 | (remote operation* adj3 robotic*).ab,kf,ti. | 11 |
| 54 | (sentiment adj3 (analys* or classif*)).ab,kf,ti. | 3113 |
| 55 | (datamining or data-mining or opinion mining).ab,kf,ti. | 17084 |
| 56 | (recursive adj3 (partitioning* or feature elimination*)).ab,kf,ti. | 5435 |
| 57 | (rough adj3 set*).ab,kf,ti. | 595 |
| 58 | "big data*".ab,kf,ti. | 18763 |
| 59 | (predictive* adj3 (analy* or model* or learn*)).ab,kf,ti. | 87039 |
| 60 | 7 or 8 or 9 or 10 or 11 or 12 or 13 or 14 or 15 or 16 or 17 or 18 or 19 or 20 or 21 or 22 or 23 or 24 or 25 or 26 or 27 or 28 or 29 or 30 or 31 or 32 or 33 or 34 or 35 or 36 or 37 or 38 or 39 or 40 or 41 or 42 or 43 or 44 or 45 or 46 or 47 or 48 or 49 or 50 or 51 or 52 or 53 or 54 or 55 or 56 or 57 or 58 or 59 | 943943 |
| 61 | 6 and 60 | 2992 |
| 62 | limit 61 to dt="20000101-20261231" | 2962 |
| **63** | **limit 62 to english** | **2935** |

| **#** | **Search Query applied in the MEDLINE database** | **Results** |
| --- | --- | --- |
| 1 | exp Sports Medicine/ | 11996 |
| 2 | Athletic Injuries/ | 33769 |
| 3 | exp Physical Therapy Modalities/ and exp Sports/ | 19504 |
| 4 | ((sport* or athletic*) and (biomechanic* or injur* or medicin* or performance* or physiolog* or recover*)).ab,kf,ti. | 85290 |
| 5 | ((sport* or athletic*) and (physiotherap* or neurophysiotherap* or physical therap* or rehab* or neurorehab*)).ab,kf,ti. | 14496 |
| 6 | 1 or 2 or 3 or 4 or 5 | 130120 |
| 7 | exp Artificial Intelligence/ | 269383 |
| 8 | ((artificial or machin* or comput* or automated) adj3 (intellig* or superintellig* or reason* or vision* or knowledge* or inferen*)).ab,kf,ti. | 122420 |
| 9 | (knowledge adj3 acquisition*).ab,kf,ti. | 5295 |
| 10 | (AI-guid* or AI-assist* or AI-aid* or AI-augment* or AI-base* or AI-system* or AI-technolog* or AI-power* or AI-process*).ab,kf,ti. | 21076 |
| 11 | ((comput* or algorithm*) adj3 heuristic*).ab,kf,ti. | 2291 |
| 12 | (hyperheuristic* or hyper-heuristic* or metaheuristic* or meta-heuristic*).ab,kf,ti. | 2883 |
| 13 | ((multicriteria or multi-criteria or "multiple criteria" or multiobjective or multi-objective or multiattribute or multi-attribute) adj3 (decision* or optimi* or algorithm*)).ab,kf,ti. | 9068 |
| 14 | MOEA.ab,kf,ti. | 267 |
| 15 | (analytic* adj3 hierarch*).ab,kf,ti. | 3233 |
| 16 | (cognitive adj3 (comput* or robotic* or technol*)).ab,kf,ti. | 4856 |
| 17 | ((machine or transfer or deep or hierarchical or labeled data or supervised or semisupervised or unsupervised or network* or Bayesian or manifold*) adj3 learn*).ab,kf,ti. | 287858 |
| 18 | ((automat* or software or system* or technol* or pattern* or optical character*) adj3 recognit*).ab,kf,ti. | 39839 |
| 19 | (back* adj3 propagation*).ab,kf,ti. | 4177 |
| 20 | "backpropagation*".ab,kf,ti. | 2982 |
| 21 | ((classificat* or detect* or learn*) adj3 algorithm*).ab,kf,ti. | 69284 |
| 22 | "classifier*".ab,kf,ti. | 53408 |
| 23 | ((confusion or error or matching) adj3 matri*).ab,kf,ti. | 2955 |
| 24 | ((feature or edge) adj3 (detect* or extract* or learn* or rank* or select*)).ab,kf,ti. | 57887 |
| 25 | (vector* adj3 (machine* or support or classif* or network* or regression*)).ab,kf,ti. | 43115 |
| 26 | (expert adj3 (system or systems)).ab,kf,ti. | 4658 |
| 27 | (fuzz* adj3 (logic* or system*)).ab,kf,ti. | 6089 |
| 28 | (hidden Markov or Markov state model*).ab,kf,ti. | 6701 |
| 29 | "iterative closest point*".ab,kf,ti. | 679 |
| 30 | (knearest neighbo* or k nearest neighbo*).ab,kf,ti. | 9650 |
| 31 | "kernel*".ab,kf,ti. | 33487 |
| 32 | (temporal difference adj3 (algorithm* or learn*)).ab,kf,ti. | 223 |
| 33 | (qlearn* or q-learn*).ab,kf,ti. | 852 |
| 34 | ((multifactor* or multi-factor*) adj3 dimension* adj3 reduction*).ab,kf,ti. | 1472 |
| 35 | (online analytic* adj3 process*).ab,kf,ti. | 63 |
| 36 | OLAP.ab,kf,ti. | 89 |
| 37 | ((outlier or anomaly) adj3 detect*).ab,kf,ti. | 5362 |
| 38 | (radial bas* adj3 function*).ab,kf,ti. | 3517 |
| 39 | (general* adj3 regression adj3 network*).ab,kf,ti. | 417 |
| 40 | "random forest*".ab,kf,ti. | 43469 |
| 41 | (knowledge base or knowledge bases).ab,kf,ti. | 12196 |
| 42 | ((biologic or biological or biomedical or gene) adj3 ontolog*).ab,kf,ti. | 42835 |
| 43 | (natural language adj3 (process* or system*)).ab,kf,ti. | 14401 |
| 44 | (language process* or NLP).ab,kf,ti. | 21514 |
| 45 | ((neural or deep or convolutional or feedforward or feed-forward) adj3 network*).ab,kf,ti. | 157883 |
| 46 | "memristor*".ab,kf,ti. | 2896 |
| 47 | "molecular dock*".ab,kf,ti. | 72795 |
| 48 | "perceptron*".ab,kf,ti. | 7759 |
| 49 | (connect* adj3 (model* or network* or system*)).ab,kf,ti. | 39414 |
| 50 | (autoencoder* or auto-encoder*).ab,kf,ti. | 7032 |
| 51 | ((companion or social or socially) adj3 (robot or robots)).ab,kf,ti. | 1417 |
| 52 | (soft adj3 (robotic* or telerobotic*)).ab,kf,ti. | 3804 |
| 53 | (remote operation* adj3 robotic*).ab,kf,ti. | 11 |
| 54 | (sentiment adj3 (analys* or classif*)).ab,kf,ti. | 3113 |
| 55 | (datamining or data-mining or opinion mining).ab,kf,ti. | 17084 |
| 56 | (recursive adj3 (partitioning* or feature elimination*)).ab,kf,ti. | 5435 |
| 57 | (rough adj3 set*).ab,kf,ti. | 595 |
| 58 | "big data*".ab,kf,ti. | 18763 |
| 59 | (predictive* adj3 (analy* or model* or learn*)).ab,kf,ti. | 87039 |
| 60 | 7 or 8 or 9 or 10 or 11 or 12 or 13 or 14 or 15 or 16 or 17 or 18 or 19 or 20 or 21 or 22 or 23 or 24 or 25 or 26 or 27 or 28 or 29 or 30 or 31 or 32 or 33 or 34 or 35 or 36 or 37 or 38 or 39 or 40 or 41 or 42 or 43 or 44 or 45 or 46 or 47 or 48 or 49 or 50 or 51 or 52 or 53 or 54 or 55 or 56 or 57 or 58 or 59 | 943943 |
| 61 | 6 and 60 | 2992 |
| 62 | limit 61 to dt="20000101-20261231" | 2962 |
| **63** | **limit 62 to english** | **2935** |
| **exp/** = term from the Medline controlled vocabulary, including terms found below this term in the MeSH hierarchy **/** = term from the Medline controlled vocabulary, does not include terms found below this term in the MeSH hierarchy **adj3** = next to each other, in any order, up to 2 word(s) in between **.ab,kf,ti**. = abstract, author keyword and title ***** at end of word = truncation of word for alternate endings | |  |

| **#** | **Search Query applied in the EMBASE database** | **Results** |
| --- | --- | --- |
| 1 | exp sports medicine/ | 20954 |
| 2 | exp sport injury/ | 40897 |
| 3 | exp physiotherapy/ and exp sport/ | 6323 |
| 4 | ((sport* or athletic*) and (biomechanic* or injur* or medicin* or performance* or physiolog* or recover*)).ab,kf,ti. | 109485 |
| 5 | ((sport* or athletic*) and (physiotherap* or neurophysiotherap* or physical therap* or rehab* or neurorehab*)).ab,kf,ti. | 20741 |
| 6 | 1 or 2 or 3 or 4 or 5 | 147811 |
| 7 | exp artificial intelligence/ | 183136 |
| 8 | exp machine learning/ | 699758 |
| 9 | exp computer vision/ | 7741 |
| 10 | exp natural language processing/ | 19764 |
| 11 | ((artificial or machin* or comput* or automated) adj3 (intellig* or superintellig* or reason* or vision* or knowledge* or inferen*)).ab,kf,ti. | 141960 |
| 12 | (knowledge adj3 acquisition*).ab,kf,ti. | 6655 |
| 13 | (AI-guid* or AI-assist* or AI-aid* or AI-augment* or AI-base* or AI-system* or AI-technolog* or AI-power* or AI-process*).ab,kf,ti. | 30068 |
| 14 | ((comput* or algorithm*) adj3 heuristic*).ab,kf,ti. | 2256 |
| 15 | (hyperheuristic* or hyper-heuristic* or metaheuristic* or meta-heuristic*).ab,kf,ti. | 2614 |
| 16 | ((multicriteria or multi-criteria or "multiple criteria" or multiobjective or multi-objective or multiattribute or multi-attribute) adj3 (decision* or optimi* or algorithm*)).ab,kf,ti. | 9705 |
| 17 | MOEA.ab,kf,ti. | 298 |
| 18 | (analytic* adj3 hierarch*).ab,kf,ti. | 3743 |
| 19 | (cognitive adj3 (comput* or robotic* or technol*)).ab,kf,ti. | 7435 |
| 20 | ((machine or transfer or deep or hierarchical or labeled data or supervised or semisupervised or unsupervised or network* or Bayesian or manifold*) adj3 learn*).ab,kf,ti. | 336338 |
| 21 | ((automat* or software or system* or technol* or pattern* or optical character*) adj3 recognit*).ab,kf,ti. | 49385 |
| 22 | (back* adj3 propagation*).ab,kf,ti. | 4780 |
| 23 | "backpropagation*".ab,kf,ti. | 3087 |
| 24 | ((classificat* or detect* or learn*) adj3 algorithm*).ab,kf,ti. | 86655 |
| 25 | "classifier*".ab,kf,ti. | 70415 |
| 26 | ((confusion or error or matching) adj3 matri*).ab,kf,ti. | 3823 |
| 27 | ((feature or edge) adj3 (detect* or extract* or learn* or rank* or select*)).ab,kf,ti. | 69148 |
| 28 | (vector* adj3 (machine* or support or classif* or network* or regression*)).ab,kf,ti. | 50528 |
| 29 | (expert adj3 (system or systems)).ab,kf,ti. | 6701 |
| 30 | (fuzz* adj3 (logic* or system*)).ab,kf,ti. | 7090 |
| 31 | (hidden Markov or Markov state model*).ab,kf,ti. | 7415 |
| 32 | "iterative closest point*".ab,kf,ti. | 859 |
| 33 | (knearest neighbo* or k nearest neighbo*).ab,kf,ti. | 11554 |
| 34 | "kernel*".ab,kf,ti. | 36740 |
| 35 | (temporal difference adj3 (algorithm* or learn*)).ab,kf,ti. | 288 |
| 36 | (qlearn* or q-learn*).ab,kf,ti. | 925 |
| 37 | ((multifactor* or multi-factor*) adj3 dimension* adj3 reduction*).ab,kf,ti. | 1731 |
| 38 | (online analytic* adj3 process*).ab,kf,ti. | 68 |
| 39 | OLAP.ab,kf,ti. | 122 |
| 40 | ((outlier or anomaly) adj3 detect*).ab,kf,ti. | 6075 |
| 41 | (radial bas* adj3 function*).ab,kf,ti. | 4184 |
| 42 | (general* adj3 regression adj3 network*).ab,kf,ti. | 456 |
| 43 | "random forest*".ab,kf,ti. | 52548 |
| 44 | (knowledge base or knowledge bases).ab,kf,ti. | 15222 |
| 45 | ((biologic or biological or biomedical or gene) adj3 ontolog*).ab,kf,ti. | 52304 |
| 46 | (natural language adj3 (process* or system*)).ab,kf,ti. | 16872 |
| 47 | (language process* or NLP).ab,kf,ti. | 25194 |
| 48 | ((neural or deep or convolutional or feedforward or feed-forward) adj3 network*).ab,kf,ti. | 185380 |
| 49 | "memristor*".ab,kf,ti. | 2483 |
| 50 | "molecular dock*".ab,kf,ti. | 81352 |
| 51 | "perceptron*".ab,kf,ti. | 8957 |
| 52 | (connect* adj3 (model* or network* or system*)).ab,kf,ti. | 49501 |
| 53 | (autoencoder* or auto-encoder*).ab,kf,ti. | 8053 |
| 54 | ((companion or social or socially) adj3 (robot or robots)).ab,kf,ti. | 1232 |
| 55 | (soft adj3 (robotic* or telerobotic*)).ab,kf,ti. | 3097 |
| 56 | (remote operation* adj3 robotic*).ab,kf,ti. | 12 |
| 57 | (sentiment adj3 (analys* or classif*)).ab,kf,ti. | 2789 |
| 58 | (datamining or data-mining or opinion mining).ab,kf,ti. | 21995 |
| 59 | (recursive adj3 (partitioning* or feature elimination*)).ab,kf,ti. | 7388 |
| 60 | (rough adj3 set*).ab,kf,ti. | 733 |
| 61 | "big data*".ab,kf,ti. | 21851 |
| 62 | (predictive* adj3 (analy* or model* or learn*)).ab,kf,ti. | 115319 |
| 63 | 7 or 8 or 9 or 10 or 11 or 12 or 13 or 14 or 15 or 16 or 17 or 18 or 19 or 20 or 21 or 22 or 23 or 24 or 25 or 26 or 27 or 28 or 29 or 30 or 31 or 32 or 33 or 34 or 35 or 36 or 37 or 38 or 39 or 40 or 41 or 42 or 43 or 44 or 45 or 46 or 47 or 48 or 49 or 50 or 51 or 52 or 53 or 54 or 57 or 58 or 59 or 60 or 61 or 62 | 1251918 |
| 64 | 6 and 63 | 3185 |
| 65 | limit 64 to dc=20000101-20261231 | 3159 |
| 66 | limit 65 to (embase or medline) | 2590 |
| **67** | **limit 66 to english** | **2555** |
| **exp/** = term from the Embase controlled vocabulary, including terms found below this term in the Emtree hierarchy **/** = term from the Embase controlled vocabulary, does not include terms found below this term in the Emtree hierarchy **adj3** = next to each other, in any order, up to 2 word(s) in between **.ab,kf,ti.** = abstract, author keyword and title *****  at end of word = truncation of word for alternate endings | | |

| **#** | **Search Query applied in the Web of Science Collection database** | **Results** |
| --- | --- | --- |
| 1 | TS=((sport* OR athletic*) AND (biomechanic* OR injur* OR medicin* OR performance* OR physiolog* OR recover*)) | 135700 |
| 2 | TS=((sport* OR athletic*) AND (physiotherap* OR neurophysiotherap* OR "physical therap*" OR rehab* OR neurorehab*)) | 17639 |
| 3 | #2 OR #1 | 139699 |
| 4 | TS=((rough NEAR/2 set*)) | 17593 |
| 5 | TS=((recursive NEAR/2 (partitioning* OR ”feature elimination*”))) | 8833 |
| 6 | TS=((datamining OR ”data-mining” OR ”opinion mining”)) | 150896 |
| 7 | TS=((sentiment NEAR/2 (analys* OR classif*))) | 29215 |
| 8 | TS=(((”remote operation*”) NEAR/2 robotic*)) | 38 |
| 9 | TS=((soft NEAR/2 (robotic* OR telerobotic*))) | 12255 |
| 10 | TS=((companion OR social OR socially) NEAR/2 (robot OR robots)) | 9009 |
| 11 | TS=((autoencoder* OR ”auto-encoder*”)) | 43164 |
| 12 | TS=((connect* NEAR/2 (model* OR network* OR system*))) | 147371 |
| 13 | TS=((perceptron*)) | 43568 |
| 14 | TS=(("molecular dock*”)) | 104249 |
| 15 | TS=((memristor*)) | 16603 |
| 16 | TS=(((neural OR deep OR convolutional OR feedforward OR ”feed forward”) NEAR/2 network*)) | 945605 |
| 17 | TS=((”language process*” OR NLP)) | 84016 |
| 18 | TS=((”natural language”) NEAR/2 (process* OR system*)) | 61972 |
| 19 | TS=(((biologic OR biological OR biomedical OR gene) NEAR/2 ontolog*)) | 47858 |
| 20 | TS=((”knowledge base” OR ”knowledge bases”)) | 43687 |
| 21 | TS=(("random forest*")) | 123925 |
| 22 | TS=((general* NEAR/2 regression NEAR/2 network*)) | 3143 |
| 23 | TS=(((”radial bas*”) NEAR/2 function*)) | 33302 |
| 24 | TS=(((outlier OR anomaly) NEAR/2 detect*)) | 64161 |
| 25 | TS=((OLAP)) | 2828 |
| 26 | TS=(((”online analytic*”) NEAR/2 process*)) | 664 |
| 27 | TS=(((multifactor* OR ”multi factor*”) NEAR/2 dimension* NEAR/2 reduction*)) | 1778 |
| 28 | TS=((qlearn* OR ”q-learn*”)) | 13378 |
| 29 | TS=((”temporal difference”) NEAR/2 (algorithm* OR learn*)) | 1160 |
| 30 | TS=((kernel*)) | 235025 |
| 31 | TS=(("knearest neighbo*" OR "k nearest neighbo*")) | 42867 |
| 32 | TS=(("iterative closest point*”)) | 3500 |
| 33 | TS=((”hidden Markov” OR ”Markov state model*”)) | 33233 |
| 34 | TS=((fuzz* NEAR/2 (logic* OR system*))) | 116332 |
| 35 | TS=((expert NEAR/2 (system OR systems))) | 39556 |
| 36 | TS=((vector* NEAR/2 (machine* OR support OR classif* OR network* OR regression*))) | 197132 |
| 37 | TS=((feature OR edge) NEAR/2 (detect* OR extract* OR learn* OR rank* OR select*)) | 513089 |
| 38 | TS=(((confusion OR error OR matching) NEAR/2 matri*)) | 18951 |
| 39 | TS=((classifier*)) | 236605 |
| 40 | TS=(((classificat* OR detect* OR learn*) NEAR/2 algorithm*)) | 325650 |
| 41 | TS=((backpropagation*)) | 21221 |
| 42 | TS=((back* NEAR/2 propagation*)) | 34741 |
| 43 | TS=(((automat* OR software OR system* OR technol* OR pattern* OR ”optical character*”) NEAR/2 recognit*)) | 174731 |
| 44 | TS=(((machine OR transfer OR deep OR hierarchical OR ”labeled data” OR supervised OR semisupervised OR unsupervised OR network* OR Bayesian OR manifold*) NEAR/2 learn*) ) | 1105857 |
| 45 | TS=((cognitive NEAR/2 (comput* OR robotic* OR technol*))) | 12785 |
| 46 | TS=((analytic* NEAR/2 hierarch*)) | 39245 |
| 47 | TS=((MOEA)) | 4620 |
| 48 | TS=((multicriteria OR ”multi-criteria” OR "multiple criteria" OR multiobjective OR ”multi-objective” OR multiattribute OR ”multi-attribute”) NEAR/2 (decision* OR optimi* OR algorithm*)) | 148070 |
| 49 | TS=((hyperheuristic* OR ”hyper-heuristic*” OR metaheuristic* OR ”meta-heuristic*”)) | 49218 |
| 50 | TS=(((comput* OR algorithm*) NEAR/2 heuristic*)) | 53404 |
| 51 | TS=((”AI-guid*” OR ”AI-assist*” OR ”AI-aid*” OR ”AI-augment*” OR ”AI-base*” OR ”AI-system*” OR ”AI-technolog*” OR ”AI-power*” OR ”AI-process*”)) | 58533 |
| 52 | TS=((knowledge NEAR/2 acquisition*)) | 20038 |
| 53 | TS=((artificial OR machin* OR comput* OR automated) NEAR/2 (intellig* OR superintellig* OR reason* OR vision* OR knowledge* OR inferen*)) | 475143 |
| 54 | #4 OR #5 OR #6 OR #7 OR #8 OR #9 OR #10 OR #11 OR #12 OR #13 OR #14 OR #15 OR #16 OR #17 OR #18 OR #19 OR #20 OR #21 OR #22 OR #23 OR #24 OR #25 OR #26 OR #27 OR #28 OR #29 OR #30 OR #31 OR #32 OR #33 OR #34 OR #35 OR #36 OR #37 OR #38 OR #39 OR #40 OR #41 OR #42 OR #43 OR #44 OR #45 OR #46 OR #47 OR #48 OR #49 OR #50 OR #51 OR #52 OR #53 | 3688603 |
| 55 | TS=(("big data*")) | 115297 |
| 56 | TS=((predictive* NEAR/2 (analy* OR model* OR learn*))) | 238724 |
| 57 | #54 OR #55 OR #56 | 3904585 |
| 58 | #57 AND #3 | 7581 |
| 59 | DOP=(2000-01-01/2026-12-31) | 64834955 |
| 60 | #58 AND #59 | 7535 |
| 61 | LA=(English) | 85303726 |
| **62** | **#60 AND #61** | **7460** |
| **NEAR/2** = next to each other, in any order, up to 2 word(s) in between **TS** = searches title, abstract, keyword plus, and author keywords *****  at end of word = truncation of word for alternate endings **DOP** = publication date **LA** = language | | |
